# Pseudo-senescence induced by palbociclib does not sensitise pleural mesothelioma cells to combinations with senolytics

**DOI:** 10.1038/s41419-026-08696-z

**Published:** 2026-04-10

**Authors:** Iswarya Sreeram, Sílvia Plans-Marin, Mabel Cruz-Rodríguez, Elisabet Aliagas, Didac Palau-Gallinat, Cristina Muñoz-Pinedo, Ernest Nadal

**Affiliations:** 1 Preclinical and experimental research in thoracic tumors (PRETT Group), Oncobell Program, Institut d’Investigació Biomèdica de Bellvitge (IDIBELL), L’Hospitalet de Llobregat (Barcelona), Spain; 2 Doctoral Programme in Medicine and Translational Research, University of Barcelona, Barcelona, Spain; 3 Doctoral Programme, Pompeu Fabra University (UPF), Barcelona, Spain; 4 Department of Medical Oncology, Catalan Institute of Oncology (ICO), ICO-HUB Comprehensive Cancer Center, L’Hospitalet de Llobregat (Barcelona), Spain; 5 Department of Clinical Sciences, Faculty of Medicine and Health Sciences, University of Barcelona, Bellvitge Healthcare Campus, L’Hospitalet de Llobregat (Barcelona), Spain

**Keywords:** Cancer, Mesothelioma

## Abstract

Pleural Mesothelioma (PM) is an aggressive neoplasm of the lung pleura with poor survival rates, highlighting the urgent need for novel therapeutic options. The CDK4/6 inhibitors abemaciclib and palbociclib have demonstrated promising results in patient-derived xenograft models of PM. In this study, we observed that palbociclib reduced proliferation, leading to increased cell size, enhanced SA-β-galactosidase activity, and elevated secretion of IL-6 and IL-8 (SASP), all of which are hallmarks of senescence. However, upon drug removal, the cells regrew. To enhance therapeutic efficacy, we attempted to induce cell death in palbociclib-pretreated PM cells with conventional senolytics, such as BH3 mimetics. While some cells showed sensitivity to Bcl-xL inhibitors, neither navitoclax nor the specific Bcl-xL inhibitor A-1331852, nor other BH3 mimetics targeting Bcl-2 (venetoclax) or Mcl-1 (S63845) increased cell death when combined with palbociclib. We explored the activity of signalling pathways after treatment with palbociclib and identified higher Src and STAT3 phosphorylation, as well as activation of the mTORC1 axis. Therefore, we employed inhibitors of these pathways, such as dasatinib, momelotinib or Torin-1, which did not synergise with palbociclib to kill the cells. In contrast, we found that the chemotherapeutic drug cisplatin induces permanent cell cycle arrest and complete senescence in PM cells. While both drugs increased the phosphorylation of γH2AX, the effects of cisplatin were stronger and more consistent across cell lines. The differential effects of palbociclib and cisplatin on permanent growth arrest were verified by sorting PM cells based on size and β-galactosidase activity. Our findings underscore the importance of understanding the nature of therapy-induced senescence when assessing the effectiveness of senolytics in different tumour models.

## Introduction

Pleural Mesothelioma (PM) is a rare, aggressive cancer of the mesothelial lining of the lung pleura, primarily caused by asbestos exposure. Germline mutations in *BAP1* and in other cancer susceptibility genes are found in 10–15% of patients with PM and are associated with an increased risk of developing the disease [1, 2]. Platinum-based chemotherapy combined with pemetrexed has been the gold standard treatment of advanced PM for decades [3]. Recently, dual checkpoint blockade with nivolumab and ipilimumab became the new standard of care in the first-line setting, having demonstrated an overall survival benefit compared with chemotherapy [4].

The limited effectiveness of available treatments underscores the urgent need for novel treatment strategies in PM. Most patients with PM (56–70%) harbour cyclin-dependent kinase inhibitor 2A (*CDKN2A)* deletions, which are associated with shorter overall survival and may predict sensitivity to CDK4/6 inhibitors [5]. In the MiST2 trial, abemaciclib demonstrated a 12-week disease control rate of 54% in patients with advanced PM who had previously received chemotherapy and whose tumours lacked p16ink4A expression [6]. Earlier work from our group demonstrated antitumour efficacy of two CDK4/6 inhibitors, palbociclib and abemaciclib, in preclinical models of PM [7]. These agents reduced cellular proliferation and caused cell cycle arrest but did not induce cell death. Instead, they increased cellular senescence in vitro, as shown by higher expression of senescence-associated markers and development of senescence-associated secretory phenotype (SASP) [8]. Although SASP can favour tumour clearance by recruiting immune cells, it may also foster chronic inflammation, tumour progression and chemoresistance [9, 10], making it a double-edged sword in cancer control.

The high frequency of *CDKN2A* deletions in PM, together with the association of CDK4 and CDK6 overexpression with shorter overall survival, and the observed antitumour activity of CDK4/6 inhibitors in preclinical models, all highlight their potential as a promising therapeutic strategy in PM. However, these treatments often induce cell cycle arrest and senescence without eliminating tumour cells [7, 11]. We hypothesised that combining CDK4/6 inhibition with either senolytic agents or inhibitors of signalling pathways upregulated upon CDK4/6 blockade could lead to higher tumour cell elimination. Here, we present our recent findings on palbociclib-induced senescence and on the efficacy of combining BH3 mimetics and other classical senolytics with palbociclib-pre-treated PM cells.

## Results

### Palbociclib-treated PM cells regrow after drug withdrawal and lose senescence features

**Fig. 1:**
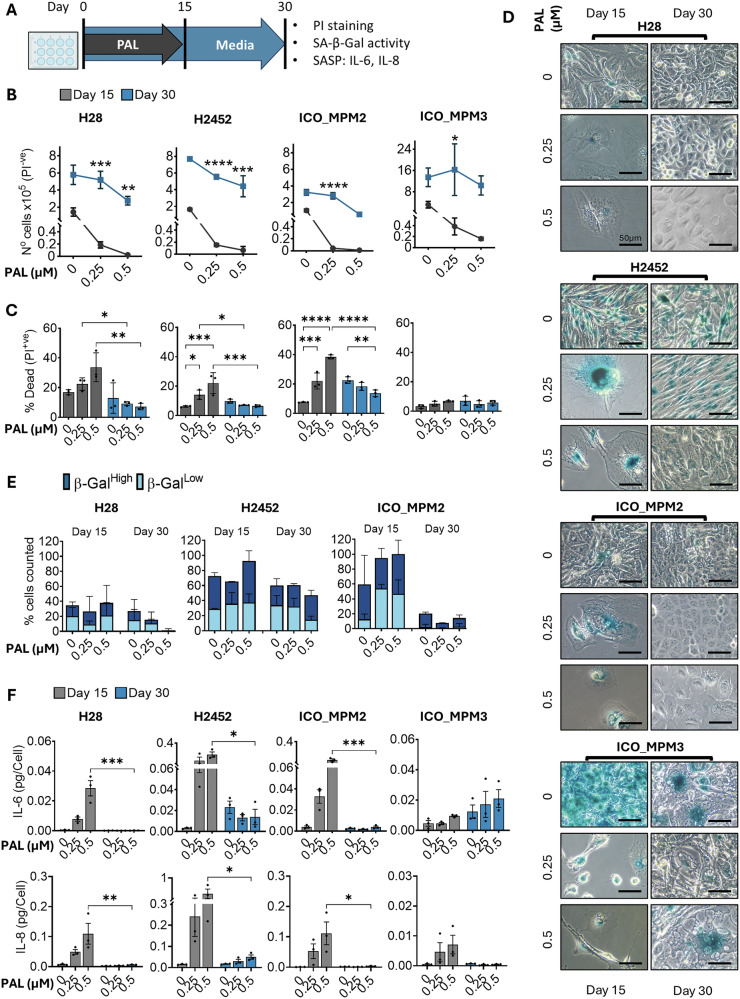
Palbociclib-treated PM cells regrow after drug withdrawal and lose senescence features. **A** Distinct PM cell lines (H28, H2452, ICO_MPM2, ICO_MPM3) were treated with palbociclib (PAL) at indicated concentrations for 15 days and allowed to grow in drug-free media for 15 additional days. **B** The total number of live cells on days 15 and 30 was counted using flow cytometry after PI staining. Data represent mean ± SD (*N* = 3). **C** The percentage of dead cells on days 15 and 30 were determined using flow cytometry after PI staining. Data represent mean ± SD (*N* = 3). **D** Representative images of PM cells stained for β-Gal activity showing cell morphology and SA-β-Gal activity on day 15 (post-treatment) and 30 (drug removal). Images were taken at 40X. **E** Supernatants collected from PM cell lines (H28, H2452, ICO_MPM2, ICO_MPM3) on days 15 and 30 were analysed for SASP factors (IL-6, IL-8). Data are presented as mean ± SEM (*N* = 3), normalised to cell numbers at day 15 and day 30. **F** The percentage of cells showing high or low SA-β-Gal activity was counted from four random images per well. Data represent mean ± SEM (*N* = 2). Where applicable, statistical significance was determined from two-way ANOVA comparing the effect on day 15 (post-treatment) to day 30 (drug removal) with multiple comparisons within each time-point (*p < 0.05, ***p < 0.001, ****p < 0.0001).

To study palbociclib-induced senescence, we treated PM cells with palbociclib at defined concentrations over an extended period, then replaced it with drug-free media. We used adenocarcinoma cell lines MCF7 (breast) and A549 (lung) as non-mesothelioma cells for comparison. Following treatment (day 15) and drug withdrawal (day 30), we tested for senescence-associated markers such as cell number, size, morphology, SA-β-Gal activity and SASP-associated cytokines (IL-6, IL-8) (Fig. 1A). To make the results more applicable, the concentrations chosen for palbociclib were close to the C_max_ in patients (0.1 μM).

Although palbociclib suppressed cell proliferation across all PM cell lines, we observed regrowth during drug removal, regardless of their sensitivity to palbociclib. The same effect was observed in A549 cells but not in MCF7 cells, which barely regrew at the highest dose used (0.5 μM). This observation highlights distinct cellular responses to palbociclib across different cell line models (Fig. 1B; SFig. 1A). Despite its strong anti-proliferative effect, palbociclib only caused a maximum of 40% cell death at 0.5 µM (Fig. 1C). In fact, in ICO_MPM3 cells, we observed minimal or no cell death. The regrowing population of cells retained sensitivity to palbociclib, as reintroducing palbociclib induced a similar anti-proliferative effect (SFig. 1B–D).

Morphological analysis revealed a pronounced enlargement of cells on day 15 post-palbociclib, indicative of a senescent-like phenotype. However, this phenotype was lost in most cell lines upon drug withdrawal, as they regrew (Fig. 1D). The only exception was MCF7 cells treated with 0.5 μM palbociclib, which retained the senescent-like morphology on day 30 (SFig. 1E).

We categorised H28, H2452, ICO_MPM2 and MCF7 cells into low- and high-intensity populations based on SA-β-Gal staining intensity. While palbociclib treatment increases SA-β-Gal activity, as evident on day 15, this effect was not sustained upon drug removal, especially in H28 and ICO_MPM2 cells, which entirely lost SA-β-Gal activity (Fig. 1E). Even in MCF7 cells that barely regrow from higher doses of palbociclib, the percentage of cells with SA-β-Gal activity was lowered upon drug removal (SFig. 1F). In H2452, ICO_MPM3 or A549, quantification of cells with high enzyme activity was challenging, possibly because high confluency in some conditions may have caused high SA-β-Gal activity.

The senescence-associated secretory phenotype (SASP) is associated with increased levels of inflammatory cytokines such as IL-6 and IL-8 [12]. Palbociclib treatment increased both IL-6 and IL-8 in all PM cells in a dose-dependent manner (Fig. 1F). However, consistent with other senescence indicators, the cytokine levels dropped after drug removal. Although these changes were not statistically significant, ICO_MPM3 sustained IL-6 levels during drug removal, but this was also observed in palbociclib-free controls.

### PM cells express anti-apoptotic proteins of the Bcl-2 family

**Fig. 2:**
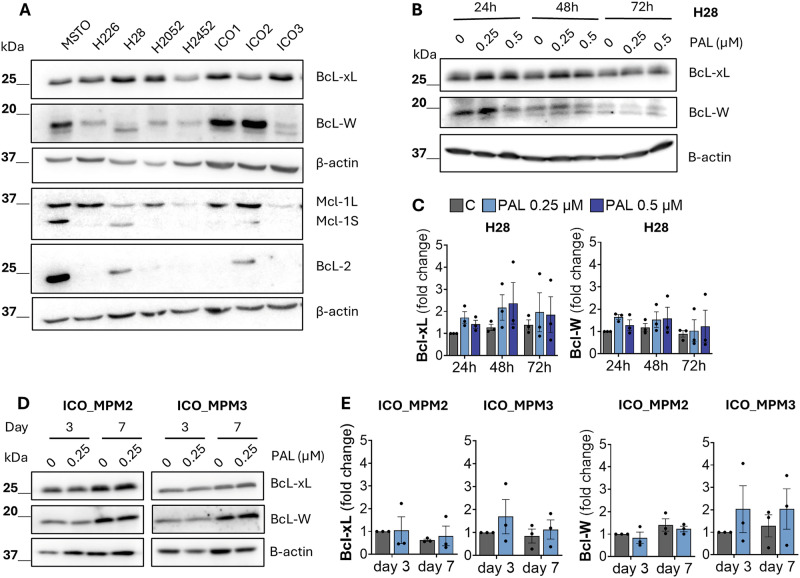
PM cells express anti-apoptotic proteins of the Bcl-2 family. **A** Representative western blots showing the baseline expression of anti-apoptotic Bcl-xL, Bcl-W, Mcl-1 and Bcl-2 in cell lysates of commercial and patient-derived PM cell lines. Blots were re-probed for β-actin as a loading control. **B** Western blots showing the expression of anti-apoptotic Bcl-xL and Bcl-W in cell lysates of H28 treated with indicated doses of palbociclib (PAL) in a time course of 24, 48 and 72 h. **C** Densitometry analysis of western blots shown in B. Data represent mean ± SEM of fold change in protein expression normalised to 24 h untreated cells (*N* = 3). **D** Western blots showing the expression of anti-apoptotic Bcl-xL and Bcl-W in cell lysates of ICO_MPM2 and ICO_MPM3 cells treated with 0.25 µM palbociclib in a time course of 3 and 7 days. **E** Densitometry analysis of western blots shown in (**C**). Data represent mean ± SEM of fold change in protein expression normalised to day 3 untreated cells (*N* = 3).

As anti-apoptotic members of the Bcl-2 family are linked to apoptotic resistance and the survival of senescent cells, we examined the baseline expression of Bcl-xL, Mcl-1, Bcl-2 and Bcl-W across our commercial and primary PM cell line models. While all PM cell lines express Bcl-xL, the expression of the rest of the anti-apoptotic proteins was highly heterogeneous (Fig. 2A; SFig. 2).

We sought to determine whether Bcl-xL or Bcl-W expression is upregulated following palbociclib treatment, which could suggest a dependency on these proteins for survival. We treated H28 cells with palbociclib at defined concentrations for 24, 48 and 72 h. Palbociclib treatment increased Bcl-xL and Bcl-W expression at all three time points compared with their untreated controls (Fig. 2B, C). As 72 h is still short, we investigated whether this trend changes with prolonged exposure to palbociclib. We treated the primary PM cell line models, ICO_MPM2 and ICO_MPM3, with palbociclib for 3 and 7 days, respectively. While the protein expression remained unaltered in ICO_MPM2 cells, ICO_MPM3 cells show a non-significant increase in both Bcl-xL and Bcl-W expression (Fig. 2D, E).

### BH3 mimetics do not synergise with palbociclib in eliminating PM cells

**Fig. 3:**
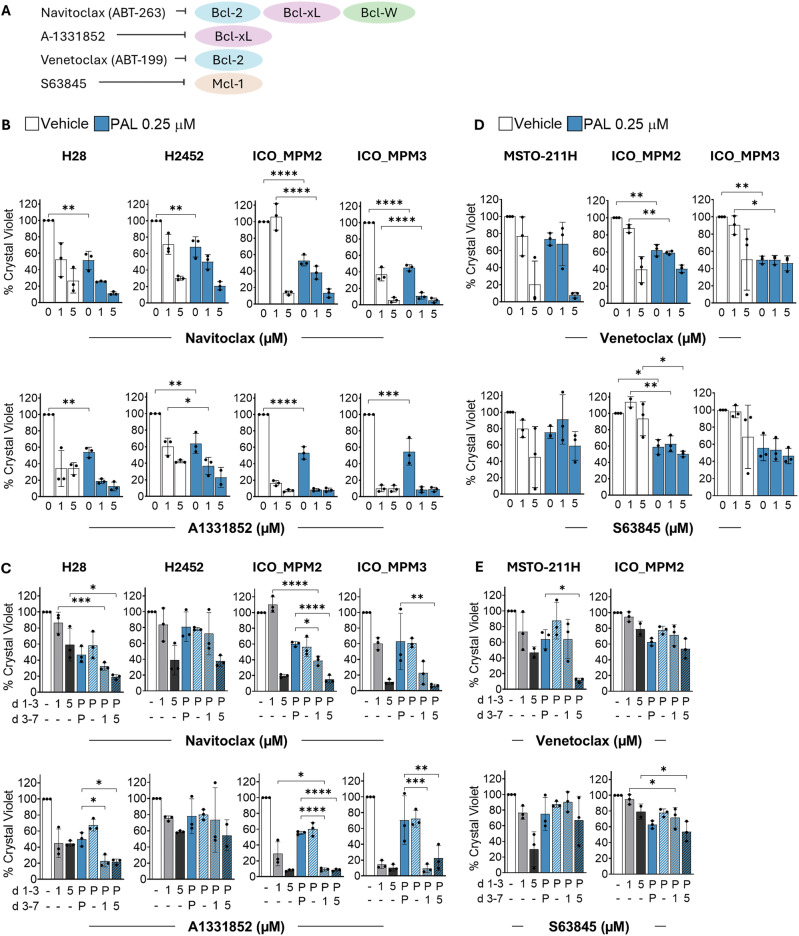
BH3 mimetics do not synergise with palbociclib in eliminating PM cells. **A** Targets BH3 mimetics used in combination with palbociclib in this study. **B** PM cell lines H28, H2452, ICO_MPM2 and ICO_MPM3 were treated with palbociclib (PAL 0.25 µM) in the presence or absence of BH3-mimetics, Navitoclax (ABT-263) or A1331852 at the indicated concentrations for 7 days. Cells were stained with crystal violet on day 7. Data represent the mean ± SD of the percentage absorbance at day 7 for crystal violet, normalised to the untreated control (*N* = 3). **C** PM cells H28, H2452, ICO_MPM2 and ICO_MPM3 were pre-treated with 0.25 µM palbociclib (‘P’) for 3 days before switching the treatment to BH3-mimetics, Navitoclax (ABT-263) or A1331852 at the indicated concentrations for 4 days more. Cells were stained with crystal violet on day 7. Data represent the mean ± SD of the percentage absorbance at day 7 for crystal violet, normalised to the untreated control (*N* = 3). **D** PM cell lines MSTO-211H, ICO_MPM2 and ICO_MPM3 were treated with palbociclib (PAL 0.25 µM) in the presence or absence of Venetoclax (A-199) or S63845 at the indicated concentrations for 7 days. Cells were stained with crystal violet on day 7. Data represent the mean ± SD of the percentage absorbance at day 7 for crystal violet, normalised to the untreated control (*N* = 3). **E** PM cells MSTO-211H and ICO_MPM2 were pre-treated with 0.25 µM palbociclib (‘P’) for 3 days before switching the treatment to Venetoclax (A-199) or S63845 at the indicated concentrations for 4 days more. Cells were stained with crystal violet on day 7. Data represent the mean ± SD of the percentage absorbance at day 7 for crystal violet, normalised to the untreated control (*N* = 3). Statistical significance determined from ordinary one-way ANOVA with multiple comparisons (*p < 0.05, **p < 0.01, ***p < 0.001, ***p < 0.0001).

We hypothesised that BH3 mimetics could induce senolysis and selectively eliminate palbociclib-pre-treated PM cells via apoptosis, as reported in other solid tumours [9, 13]. To test this, we studied both concurrent and sequential treatment strategies, combining palbociclib with BH3 mimetics targeting different anti-apoptotic members of the Bcl-2 family (Fig. 3A).

Our results showed that all PM cell lines were sensitive to Bcl-xL inhibition by navitoclax/A-1331852 at the tested concentrations. However, concurrent treatment with palbociclib did not significantly enhance this effect, except in H28, H2452 and H226 cells, where the combination appears to be additive (Fig. 3B, SFig. 3A). Interestingly, H2452 and H226 cells which were modestly sensitive to concurrent administration of palbociclib and navitoclax/A-1331852, were resistant to this combination when administered sequentially (Fig. 3C, SFig. 3B). These findings suggest that in cells already sensitive to Bcl-xL inhibition, palbociclib does not increase sensitivity, indicating the response is due to intrinsic Bcl-xL dependency, not palbociclib-induced senescence. In contrast, we did not observe any significant effects when combining palbociclib with S63845 or venetoclax (Fig. 3D, E, SFig. 3C, D). All PM cell lines remained consistently resistant to these combinations, except MSTO-211H, which showed a slightly enhanced effect on venetoclax combined with palbociclib.

### Adaptive response to palbociclib guided other combination treatments

**Fig. 4:**
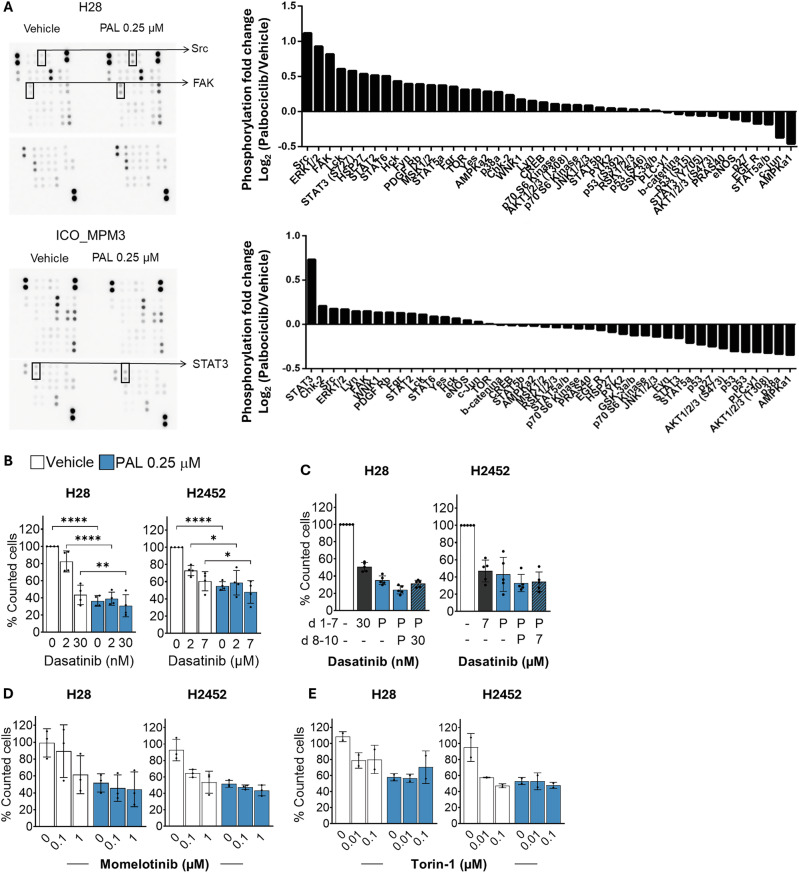
Adaptive response to palbociclib guided other combination treatments. **A** H28 and ICO_MPM3 cells were treated with palbociclib (PAL 0.25 µM) for 3 days and checked for the phosphorylation and activation of kinases in a phospho-protein array. **B** H28 and H2452 cells were concurrently treated with palbociclib (PAL 0.25 µM) in the presence or absence of Dasatinib (Src kinase inhibitor) for 7 days. On day 7, cells were counted from three random pictures per well following crystal violet staining. Data represent mean ± SD (*N* = 4). **C** H28 and H2452 cells were pre-treated with palbociclib (PAL 0.25 µM) for 7 days before switching the treatment to dasatinib/drug-free media for 3 more days (sequential). On day 10, cells were counted from three random pictures per well following crystal violet staining. Data represent mean ± SD (*N* = 5). H28 and H2452 cells were concurrently treated with palbociclib (PAL 0.25 µM) in the presence or absence of Momelotinib (JAK-STAT kinase inhibitor) (**D**) or Torin-1 (mTOR inhibitor) (**E**) for 3 days. On day 3, cells were counted from three random pictures per well following crystal violet staining. Data represent mean ± SD (*N* = 3 for Momelotinib; *N* = 2 for Torin-1).

Since BH3 mimetics did not synergise with palbociclib to cause cell death in PM, we explored alternative strategies to eliminate palbociclib-treated cells. A phospho-kinase protein array performed on PM cell lines treated with palbociclib for 72 h revealed the increased phosphorylation of key proteins involved in cell proliferation and survival, especially of Src kinases, STAT3 (target of JAK kinases) and mTOR (Fig.4A; SFig. 4A). We tested whether inhibiting these kinases with their respective inhibitors could eradicate palbociclib-treated PM cells.

Similar to BH3 mimetics, we evaluated dasatinib (Src kinase inhibitor) in combination with palbociclib using both concurrent (Fig. 4B) and sequential treatment (Fig. 4C) in H28 and H2452 cell lines. At concentrations near the IC50, dasatinib did not synergise with palbociclib in either cell line. However, we observed that combining higher doses of dasatinib with palbociclib appears to alter the enlarged morphology typical of palbociclib (SFig. 4B). Whether these changes are related to the senescent phenotype requires further investigation.

We also tried combining palbociclib with the JAK1/JAK2 inhibitor momelotinib (CYT-837) (Fig. 4D) and the mTORC1/C2 inhibitor Torin-1 (Fig. 4E) in H28 and H2452 cells. However, neither combination demonstrated a significant antitumour effect in PM models. Based on previous studies that show palbociclib-induced cell enlargement increases mitochondrial activity [14], we tested the mitochondrial complex I inhibitor metformin in combination with palbociclib (SFig. 4C, D). We also identified MKC8866, an IRE1α kinase inhibitor, as a compound of interest by analysing publicly available data from palbociclib-treated PM cells [8]. However, none of these combinations showed efficacy in PM cells when combined with palbociclib (SFig. 4E).

### Cisplatin induces a persistent senescent-like phenotype in PM cells

Since classical senolytics and other compounds failed to sensitise palbociclib-pretreated PM cells to cell death, we questioned the nature of palbociclib-induced senescence by comparing it with that induced by cisplatin and by increasing doses. PM cell lines (H28, H2452) and MCF7 cells were treated with 10x higher doses of palbociclib (5 µM) or cisplatin (25 µM) for 24 h and switched to drug-free media for 9 days (SFig. 5A). Even at a higher dose, palbociclib failed to induce a permanent growth arrest in PM cells. In contrast, cisplatin treatment produced a potent anti-proliferative effect with no regrowth upon drug withdrawal (SFig. 5B).

**Fig. 5:**
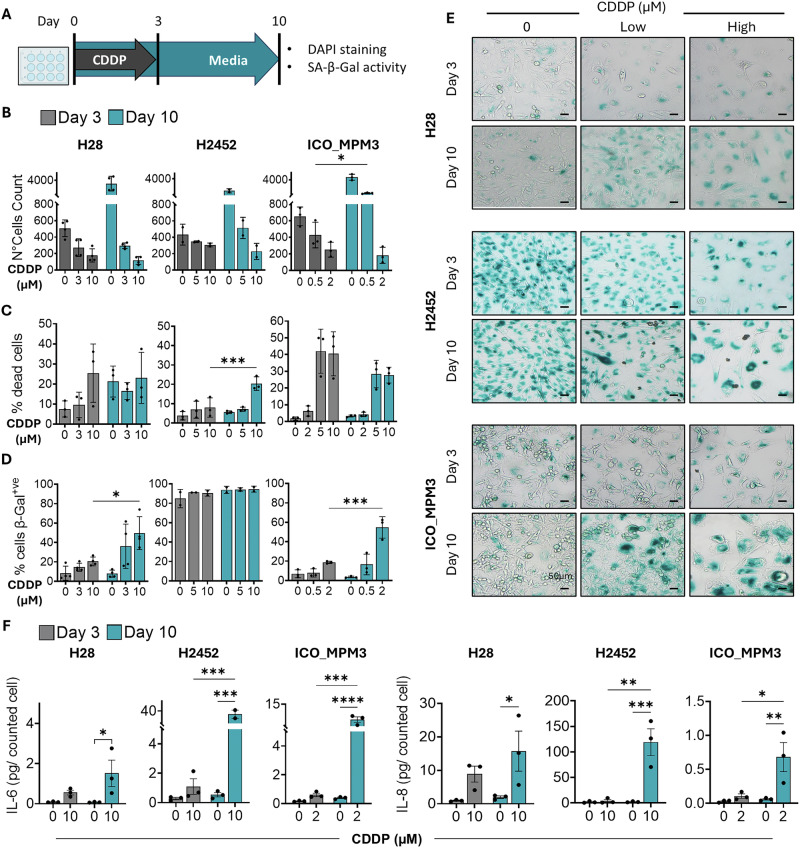
Cisplatin induces a persistent senescent-like phenotype in PM cells. **A** PM cell lines H28, H2452 and ICO_MPM3 were treated with the indicated doses of cisplatin (CDDP) for 3 days, followed by 7 days in drug-free media. **B** On days 3 and 10, cell nuclei were counted from 3 random pictures per well following DAPI staining. Data represent the average number of cells counted for each condition, mean ± SD (*N* = 4 for H28, *N* = 3 for H2452, ICO_MPM3). **C** The percentage of dead cells on days 3 and 10 at given doses of cisplatin was determined using flow cytometry after PI staining. Data represent mean ± SD (*N* = 3). **D** The percentage of cells counted positive for β-Gal activity on day 3 and on day 10. Data represented as mean ± SD (*N* = 4 for H28; *N* = 2 for H2452, *N* = 3 for ICO_MPM3). **E** Representative images of PM cell lines stained for SA-β-Gal activity taken at 10X on day 3 and day 10. **F** PM cell lines (H28, H2452, ICO_MPM3) were treated with cisplatin (CDDP) for 3 days and then grown in drug-free media for 7 more days. Supernatants collected on days 3 and 10 were analysed for SASP factors (IL-6, IL-8). Data are shown as mean ± SEM (*N* = 3), normalised to the number of cells counted for each condition. In all cases, statistical significance was determined using two-way ANOVA with multiple comparisons, comparing the effect on day 3 vs that on day 10 (*p < 0.05, **p < 0.01, ***p < 0.001).

Because these doses of cisplatin were above clinically achievable concentrations, we conducted an additional experiment with longer exposures and with doses below the maximum tolerated dose (C_max_) in patients, which corresponds to 14.4 µM in vitro [15]. We used a dose close to each cell individual IC50 and an arbitrary higher dose [7], all below 14 µM. Senescence-associated markers were analysed at day 3 (post-treatment) and at day 10 (post-washout) (Fig. 5A). We observed that cisplatin-pretreated cells did not resume proliferation upon drug removal (Fig. 5B). Notably, the average cell counts for cisplatin-pretreated cells on day 10 were lower than those on day 3, suggesting a sustained effect upon drug removal. Growth between day 10 and day 3 was compared between cisplatin and palbociclib, and the effect of cisplatin was more pronounced, even at low doses (SFig. 6A). We wondered whether this effect was due to cisplatin-induced cytotoxicity and assessed cell death. Although higher doses of cisplatin induced some cell death, the overall percentage remained below approximately 40% (Fig. 5C, SFig 6B, C), suggesting that the effect was mainly cytostatic. Cisplatin-induced cell death on day 3 was comparable to that of palbociclib (SFig. 6B). Cells pretreated with cisplatin exhibited an enlarged phenotype and increased SA-β-Gal activity on day 3 (Fig. 5D, E). Moreover, enlargement and β-Gal activity were intensified in H28 and ICO_MPM3 cells after drug removal, indicating a robust senescence phenotype, although inherently high β-galactosidase expression made this evaluation challenging in H2452 cells. We also observed that the SASP associated with cisplatin treatment was sustained and even elevated after drug removal (Fig. 5F). All these data qualify cisplatin as a bona fide inducer of senescence in PM cells.

### Single-cell sorting of live senescent cells demonstrated regrowth in palbociclib-treated cells

Since the cell population after palbociclib treatment is highly heterogeneous, we aimed to determine whether the regrowth arises from the small fraction of SA-β-Gal^Low^ cells -that might still be proliferating- or from the SA-β-Gal^High^ cells that may be permanently arrested and potentially senescent. For this, we performed a senescent cell sorting experiment using spectral cytometry.

**Fig. 6:**
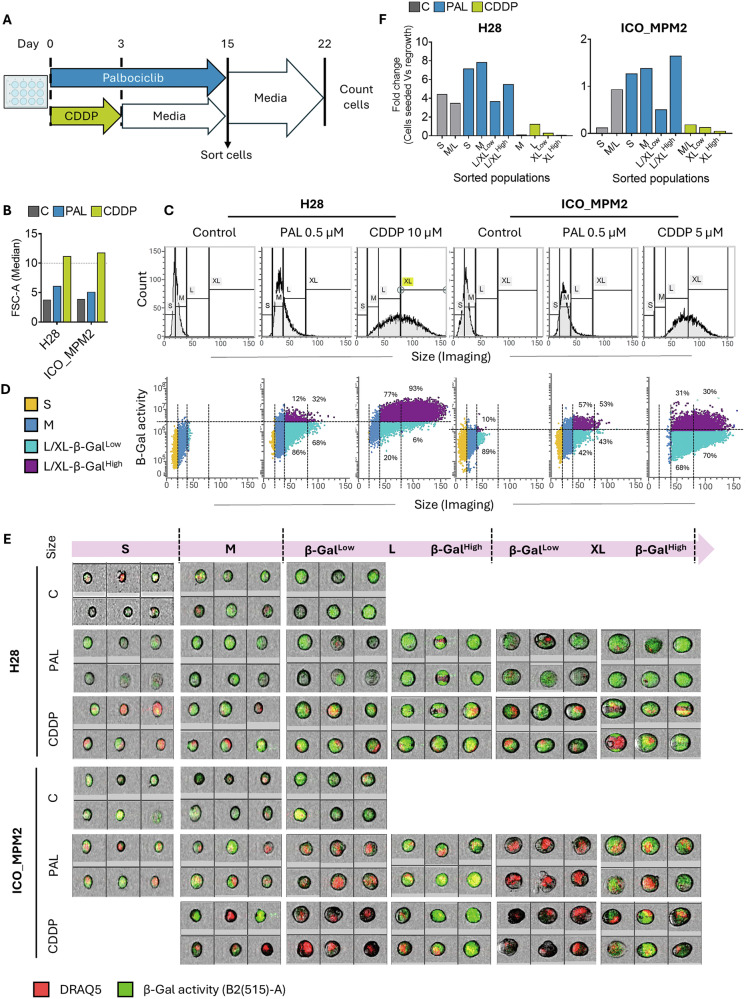
Single-cell sorting of live senescent cells demonstrated regrowth in palbociclib-treated cells. **A** PM cell lines H28 and ICO_MPM2 were treated with 0.5 µM palbociclib (PAL) for 15 days. As a positive control for permanent cell cycle arrest, cells were treated with Cisplatin (CDDP) for 3 days, then cultured in drug-free media for 12 days. On day 15, surviving cells were stained with DAPI, DRAQ5 and SA-β-Gal substrate, then sorted by size and SA-β-Gal activity. The sorted populations were re-cultured in drug-free media for 7 days and evaluated for viability. **B** Quantification of FSC in H28 and ICO_MPM2 cells treated with palbociclib or cisplatin on day 15. The y-axis represents Median-FSC (x10^5^) from total singlets gated positive for DRAQ5. **C** Histograms showing differences in size between control and cells treated with palbociclib or cisplatin on day 15. **D** Dot plots showing the population distribution and SA-β-Galactosidase activity of cells on day 15, before sorting. The numbers represent the percentage of cells with high or low SA-β-Gal activity within the parent population of large (L) or extra-large (XL) cells. **E** Representative images from BD S8 cell sorter with BD Cell view^TM^ showing the size and SA-β-Gal activity in H28 and ICO_MPM2 cells on day 15 before sorting. **F** The sorted populations of H28 and ICO_MPM2 cells were re-cultured in drug-free media for 7 days, and the number of live cells was counted by flow cytometry. Data are presented as fold changes between the initial number of cells sorted into each sub-population on day 15 and the number of live cells counted for each sub-population on day 22. A fold change >1 represents regrowth.

PM cell lines H28 and ICO_MPM2 underwent long-term palbociclib treatment (0.5 µM) and were subjected to single-cell sorting based on cell size and SA-β-Gal activity (SFig. 7G, H). PM cells pre-treated with cisplatin and allowed to senesce in drug-free media served as a positive control for cell senescence. After sorting, the cell populations were reattached and cultured for 7 days, then collected and counted to assess proliferation (Fig. 6A).

As shown in previous figures, cells treated with palbociclib or cisplatin increased in size, although cells with cisplatin pre-treatment were significantly bigger than those with palbociclib treatment (Fig. 6B). This is also evident when comparing the percentage of cells in the ‘L’ or ‘XL’ sub-populations when cells were grouped by size measured by FACS/imaging (Fig. 6C, SFig. 7I). H28 cells exhibited higher SA-β-Gal activity than ICO_MPM2 cells (SFig. 8J, Fig. 6D). We also observed that both cell size and SA-β-Gal activity tend to increase across the entire cell population in response to palbociclib or cisplatin, suggesting that senescence might not be an isolated event in a specific subgroup of cells (Fig. 6C–E, SFig. 8A). Sorted cells from the palbociclib-pre-treated population were seeded and regrew regardless of cell size or SA-β-Gal activity (Fig. 6F). In contrast, almost none of the sorted populations from cisplatin-pre-treated cells regrew. These results, therefore, challenge the notion of palbociclib-induced senescence.

### Differential expression of canonical senescence markers in cisplatin- and palbociclib-treated PM cells

**Fig. 7:**
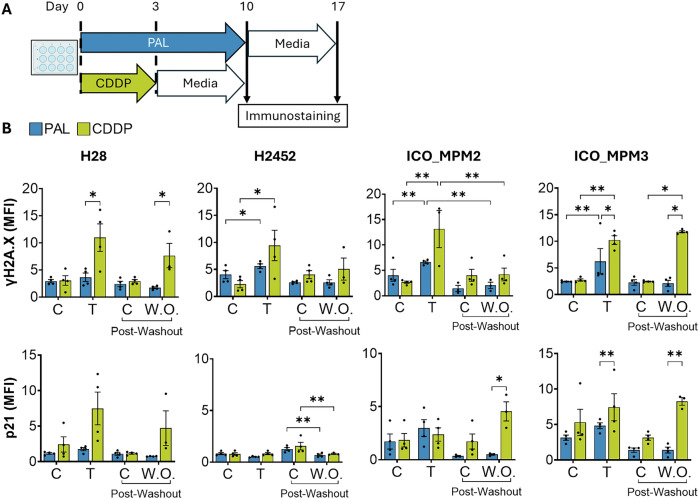
Differential expression of canonical senescence in cisplatin- and palbociclib-treated PM cells. **A** PM cells H28, H2452, ICO_MPM2 and ICO_MPM3 were treated with 0.25 µM of palbociclib (PAL) for 10 days before switching to drug-free media for 7 more days. For cisplatin (CDDP), cells were treated for 3 days, then switched to drug-free media for 7 more days. At all times, cells were collected and immunostained to observe the expression of ɣ-H2A.X and p21 through flow cytometry. **B** Data showing Median Fluorescent Intensity (MFI) for ɣ-H2A.X and p21 on days after treatment (T) with palbociclib (day 10) and cisplatin (day 3) compared to post-washout. Data represent mean ± SEM from at least 3 biological replicates. Statistical significance was evaluated using Two-Way ANOVA comparing MFI across groups for Control (C), Treatment (T) and Washout (W.O.) (*p < 0.05, ***p < 0.001, ****p < 0.0001).

To further clarify the differences observed between palbociclib- and cisplatin-induced senescence, we tested the expression of phospho-γH2AX and p21, which indicate DNA damage and cell-cycle arrest, respectively. PM cells were treated with palbociclib or cisplatin for the indicated number of days, then switched to drug-free media for a week (Fig. 7A). Palbociclib induced γH2AX phosphorylation in most cell lines, while the increase in p21 was not significant (Fig. 7B). Both phospho-γH2AX and p21 levels were higher during cisplatin treatment compared to palbociclib in all cells tested, except for p21 in H2452, which was not increased by any treatment (Fig. 7B). Any minor increase in protein expression induced by palbociclib was consistently reversed upon drug withdrawal. However, cisplatin-pre-treated cells maintained the levels of both markers upon drug withdrawal, confirming that cisplatin exerts a stronger, more persistent effect.

## Discussion

PM is a rare and aggressive cancer with limited therapeutic options. All PM cell lines used in this study, except H226, have *CDKN2A* deletions and consequently, p16 expression is lost in all of them, including H226 [7], which may have lost it for other reasons. p16 loss has been associated with sensitivity to CDK4/6 inhibitors [8], which provided a rationale for selecting palbociclib as a potential therapeutic strategy in PM. We observed that at clinically relevant doses, palbociclib effectively halts cell proliferation; however, as previously noted, this cytostatic effect appears reversible, as palbociclib-pretreated PM cells resume growth upon drug withdrawal [16]. Some cell lines (H2452, ICO_MPM3) exhibited more pronounced regrowth than others (H28, ICO_MPM2), warranting further investigations into the biological factors underlying these differential responses.

Despite regrowth, PM cells remained sensitive to palbociclib reintroduction, indicating that the drug induces a reversible cell cycle arrest that can be reinforced. These findings contrast with a study that showed that some PM cell lines (H2452, MSTO-211H) developed resistance after prolonged exposure. They also report cell cycle arrest was largely irreversible at 1 µM palbociclib, even though proliferation-associated genes reactivate 48 h after drug washout [8]. This raises the possibility that irreversible growth arrest may only be achievable at higher, clinically limiting doses.

Senescence is considered an irreversible state of growth arrest, although emerging evidence suggests it may be reversible in some contexts. While palbociclib induces a senescent-like phenotype in PM cells, the complete reversal of cell enlargement, β-Gal activity and SASP (IL-6, IL-8) during regrowth challenges the notion that palbociclib triggers bona fide senescence. Nevertheless, these findings underscore the importance of eliminating these cells to prevent tumour progression and improve therapeutic efficacy in PM.

The anti-apoptotic Bcl-2 family members are associated with apoptotic resistance in senescent cells, and BH3 mimetics have demonstrated senolytic activity across distinct tumour models [17, 18]. While most PM cells express these proteins at baseline levels, we could not establish a clear relationship between protein expression and sensitivity to BH3-mimetics. For example, H226 cells expressed Mcl-1 but remained insensitive to S63845. MSTO-211H was the only cell line in which Bcl-2 expression correlated with sensitivity to venetoclax. Independent of Bcl-xL expression, all PM cell lines were sensitive to single-agent navitoclax and A1331852, with the strongest responses observed with A1331852, the more selective Bcl-xL inhibitor. For instance, ICO_MPM2 expressed relatively low Bcl-xL yet was among the most sensitive cell lines to Bcl-xL inhibition. These findings suggest that factors beyond protein expression modulate sensitivity to Bcl-xL inhibitors, while still supporting the previously reported dependency of PM cells on Bcl-xL [19, 20].

BH3 mimetics targeting different anti-apoptotic proteins have previously demonstrated senolytic potential by selectively eliminating senescent cells [9, 21, 22]. In H28 and ICO_MPM3 cells, palbociclib treatment slightly increases Bcl-xL and Bcl-W expression, suggesting a possible correlation between palbociclib-induced cell cycle arrest and anti-apoptotic protein expression. Unexpectedly, in PM cells, we observed only a modest effect in combining palbociclib with BH3 mimetics, even with navitoclax, which is a well-established senolytic agent that selectively eliminates senescent cells [23]. Instead, we found that sensitivity to navitoclax/A1331852 in combination treatments is driven by an intrinsic dependence on Bcl-xL in the non-senescent state, rather than by palbociclib-induced senescence. A recent study reported a similar pattern of navitoclax senolytic activity across other cell line models [24].

Surprisingly, MSTO-211H cells were resistant to the effects of navitoclax and S63845 when administered sequentially after palbociclib, indicating that palbociclib does not prime these cells for apoptosis. A recent study similarly showed that palbociclib fails to sensitise PM patient-derived xenograft (PDX) tumours to BH3-mimetic-induced apoptosis, particularly with navitoclax [25]. Although Bcl-xL inhibition may increase cellular dependency on Mcl-1 [26], further investigation is needed to determine whether palbociclib leverages this effect to promote survival or resistance to navitoclax in PM cells.

The upregulation and hyperactivation of the Src family of kinases and STAT3 observed in our phospho-kinase array have also been previously reported in various mesothelioma tumour samples [27]. After DNA damage, Src kinase activation can also shift cell fate from apoptosis to senescence [28]. Given the upregulation of Src upon palbociclib treatment, we deemed it an attractive target, but combining palbociclib with the Src kinase inhibitor dasatinib (BMS354825) did not elicit senolytic effects, nor did it induce significant changes in cell proliferation in H28 or H2452 cells. Additionally, combining palbociclib with inhibitors of other key signalling pathways, such as momelotinib or Torin-1, did not significantly reduce cell numbers, suggesting that these pathways may not be directly involved in the survival of PM cells treated with palbociclib.

To examine the nature of palbociclib-induced senescence, we compared its effects with those of cisplatin, a well-established inducer of senescence. The fact that cisplatin induced permanent cell cycle arrest in PM cells with senescence-like features validates its potential to induce canonical senescence compared to palbociclib. Cell death induced by either cisplatin or palbociclib at 3 days remained below 40% in all cell lines, suggesting that both drugs exert a predominantly cytostatic effect. Combinations of cisplatin and BH3 mimetics have previously demonstrated efficacy in cell models of PM [19]. However, this effect is likely to result from enhancement of cell death and needs further investigation .

Understanding the nature of therapy-induced senescence is essential for interpreting regrowth and predicting sensitivity to senolytic drug combinations. While several studies have reported that therapy-induced senescence may be reversible [29, 30], the lack of specific and definitive biomarkers for senescent cells makes this determination challenging. In this context, what is often termed ‘reversible senescence’ could arguably be more accurately classified as ‘pseudo senescence’, defined as a state of cell cycle arrest accompanied by senescence-associated features such as SASP induction, cell enlargement and increased SA-β-Gal activity.

SA-β-Gal is a widely used marker of senescence, but it is indeed not exclusive to this state and can also be observed in cells undergoing stress-induced cell cycle arrest [12]. Nonetheless, we attempted to sort live palbociclib or cisplatin-pre-treated PM cells based on size and SA-β-Gal activity using spectral cytometry, which enabled us to visualise the cells and facilitate image-based spectral sorting. When comparing palbociclib and cisplatin treatments, cell size appeared to correlate more closely with the degree of senescence than SA-β-Gal activity. However, neither of these markers reliably predicted subsequent regrowth. Assuming that senescence is confined to large, β-Gal–positive cells, the smaller, β-Gal–low fraction would be expected to retain proliferative capacity and drive subsequent population regrowth. Instead, all subpopulations from palbociclib-pre-treated PM cells regrew, regardless of cell size or β-Gal activity. In contrast, none of the subpopulations from cisplatin-pre-treated cells regrew. Due to the technical complexity and extended workflow of this novel assay, we report a single biological replicate for each cell line, which show similar results in both lines and is consistent with our hypothesis that cisplatin induces a more stable and pronounced senescence state than palbociclib.

Prolonged treatment with CDK4/6 inhibitors has been shown to induce high genotoxicity in distinct cancer cell lines, and the ability of palbociclib to induce cell cycle arrest and senescence was linked to DNA damage signalling proteins such as p53, p21 and p16 [11, 31–33]. Palbociclib was also shown to promote p53 degradation via the AKT-MDM2 axis, thereby inhibiting p53-dependent p21 accumulation to promote complete senescence in PM [16]. We observed that the expression of the DNA damage-associated marker, phospho-ɣH2AX and the cell cycle arrest marker, p21, was significantly lower in palbociclib pre-treated PM cells than in cisplatin-treated cells. However, the role of p21 in palbociclib-induced senescence remains debatable, as palbociclib can induce cell cycle arrest through p21-independent mechanisms [34]. The nature of the DNA damage triggered by palbociclib and the molecular determinants that enable PM cells to escape from palbociclib cell cycle arrest warrant further investigation.

Despite the incorporation of immunotherapy into the therapeutic armamentarium for PM, long-term survival remains limited for most patients. CDK4/6 inhibitors have demonstrated therapeutic efficacy in various tumour models, especially in metastatic breast cancer [35–38] and more modestly in mesothelioma [6]. In clinical practice, CDK4/6 inhibitors are typically administered on a 3-week-on/1-week-off schedule. Our findings indicate that the cytostatic effects of palbociclib are largely reversible and short-lasting, as arrested cells regain proliferative capacity after drug withdrawal. This observation raises important questions about palbociclib’s potential to induce true senescence, as palbociclib-treated cells were not sensitised to cell death when combined with senolytic agents such as ABT-263 (navitoclax) or dasatinib. If palbociclib instead induces a state of ‘pseudo-senescence’ in mesothelioma, this may ultimately limit the clinical efficacy of CDK4/6 inhibition by facilitating the risk of tumour regrowth or progression. Collectively, our findings underscore the critical importance of defining the nature of therapy-induced senescence when evaluating the therapeutic potential of senolytics strategies in any tumour model.

## Materials and methods

### Cell culture and cell lines

Five commercial PM cell lines, MSTO-211H, H28, H226, H2052 and H2452, and adenocarcinoma cell lines MCF7 (Breast) and A549 (lung) were purchased from ATCC (Manassas, VA). Primary PM cell lines, ICO_MPM1, ICO_MPM2 and ICO_MPM3, were derived from pleural effusions obtained from three patients with PM. ICO_MPM2 was derived from a chemotherapy-naïve patient, while ICO_MPM1 and ICO_MPM3 were from patients who progressed on platinum and pemetrexed [7]. All commercial cell lines and ICO_MPM3 were cultured in RPMI 1640 medium-Glutamax™ supplemented with 10% FBS and 1% penicillin/streptomycin. For ICO_MPM1 and ICO_MPM2, the above-mentioned medium was further supplemented with 1% Glutamax, 1% HEPES, 0.2% Insulin, 0.1% Transferrin (10 mg/ml) and 0.1% ACL4 (Prepared with 25 nM Sodium selenite, 50 nM Hydrocortisone, 0.16 nM EGF, 0.01 mM Ethanolamine, 0.01mM O-phosphoryl ethanolamine, 0.1 nM 3,3´,5-triiodo-L-thyronine and 0.5 mM Sodium pyruvate) [39]. All cell lines were incubated and maintained at 37 °C with 5% CO2 and were routinely tested for mycoplasma contamination.

### Antibodies and compounds

Antibodies against Bcl-xL (#2764) and Bcl-W (#2724) were purchased from Cell Signalling Technology (Danvers, MA). Bcl-2 (sc509) and Mcl-1 (sc819) are from Santa Cruz Biotechnology (Dallas, TX) and β-Actin (MAB1501R) was obtained from Merck Millipore (Burlington, MA). For immunostaining, antibodies for γH2A.X (phospho-Ser139) (#2577) and p21 Waf1/Cip1 (12D1) (#2947) were purchased from Cell Signalling Technology (Danvers, MA). Anti-rabbit AF488 and AF546 were purchased from Life Technologies (A11008) and Invitrogen (A11010), respectively. Palbociclib (PD0332991), Torin-1 and dasatinib (BMS-354825) were purchased from Selleckchem (Houston, TX). Navitoclax (ABT-263), venetoclax (ABT-199), S63845 (Mcl-1 inhibitor) and A1331852 (Bcl-xL inhibitor) were purchased from MedChemExpress (New Jersey, USA). Momelotinib was purchased from Invivogen (San Diego, California, USA). Metformin hydrochloride was purchased from Sigma. MKC8866 was a gift from E. Chevet. DRAQ5 was a gift from Oscar Fornas, PRBB. Cisplatin was purchased at the Pharmacy of the Catalan Institute of Oncology (ICO, L’Hospitalet de Llobregat, Barcelona, Spain).

### Western blot analysis

Whole-cell lysates from PM cells were collected under untreated conditions to assess baseline levels of anti-apoptotic Bcl-2 family proteins. For the time course, H28 cells were seeded at 0.4 × 10^6^ and left to attach overnight before palbociclib treatment (0.25 µM, 0.5 µM) for 24, 48 and 72 h. ICO_MPM2 and ICO_MPM3 cells were seeded at 0.1 × 10^6^ and treated with 0.25 µM palbociclib for 3 and 7 days. Whole-cell lysates were collected at each time point after treatment, and western blots were done to check the expression of Bcl-xL and Bcl-W as described previously [40].

### Crystal Violet cell staining/counting

PM cells treated with combinations of palbociclib and the respective senolytics were fixed with 4% formaldehyde, then stained with crystal violet for 20 min at RT. The plate was dried overnight, and crystal violet was solubilised using a 10% SDS solution. Absorbance was read at 570 nm to measure cell viability. For cell counting experiments, the number of cells was counted from 3–4 random pictures per well, taken after staining with crystal violet.

### Cell counting with DAPI

For cell counting with DAPI staining, PM cells were fixed with 4% formaldehyde for 15 min at RT and stained with DAPI for an hour, protected from light. DNA was visualised with a Zeiss Axio Observer Z1+Apotome inverted fluorescent microscope provided by IDIBELL Scientific and Technical Services. Cell nuclei were counted using the Stardist2D plugin in Fiji (ImageJ) software.

### Measurement of cellular senescence by β-Galactosidase activity

Senescence was measured by the procedure provided in the Senescence β-Galactosidase Staining Kit (#9860 Cell Signalling Technologies). For live senescent cell sorting, the Cellular Senescence Live Cell Analysis Assay Kit (ENZ-KIT130) was purchased from Enzo Life Sciences. The staining protocol was followed according to the manufacturer’s guidelines. Cells positive for β-Gal activity were categorised into β-Gal^Low^ and β-Gal^High^ through visual inspection and were manually counted using ImageJ software.

### Flow cytometry measurement of cell death

PM cells treated with cisplatin or palbociclib were harvested and stained with Propidium iodide (PI) to measure cell death. Samples were acquired on a CytoFLEX flow cytometer (Beckman Coulter Life Sciences), provided by IDIBELL Scientific and Technical Services. Data were analysed using CytExpert software.

### ELISA

Supernatants were collected after palbociclib or cisplatin treatments, and levels of IL-6 and IL-8 in cell culture supernatants were measured using human IL-6 and IL-8 ELISA kits (R&D Systems, Minneapolis, MN, USA) according to the manufacturer’s instructions.

### Long-term palbociclib treatment and drug removal

PM cell lines H28, H2452, ICO_MPM2 and ICO_MPM3, adenocarcinoma cell lines MCF7 (Breast) and A549 (lung) were seeded at low densities and allowed to attach for 24 h before palbociclib treatment (0.25 µM, 0.5 µM). Palbociclib treatment was maintained for 15 days, with the drug replenished every 3–4 days. At 15 days, the treatment was stopped, and the cells were allowed to grow in drug-free media for 15 additional days. At both endpoints (days 15 and 30), cell viability, cell death, IL-6 and IL-8 levels and SA-β-Gal activity were measured. The breast cancer cell line, MCF7 and the lung adenocarcinoma cell line, A549, were used as non-mesothelioma controls.

### Cisplatin treatment and drug removal

PM cell lines H28, H2452 and ICO_MPM3 were treated with Cisplatin (0.5–10 µM) for 3 days. On day 3, the treatment was stopped, and the cells were left to grow in drug-free media for 7 more days. Controls were reseeded to prevent over-confluence. On days 3 and 10, cell viability, cell death, IL-6 and IL-8 levels and SA-β-Gal activity were measured.

### Combination treatments with palbociclib

Two modes of treatment were followed: concurrent and sequential. For palbociclib combinations with BH3 mimetics or Dasatinib, PM cells were treated individually with 0.25 µM palbociclib, BH3 mimetics (1 µM, 5 µM)/dasatinib (0.002–7 µM), or a combination of both drugs at the indicated concentrations for 7 days. For combination treatments of palbociclib with metformin (0.1–50 µM), Torin-1 (0.01–0.1 µM), momelotinib (0.1–1 µM), or MKC8866 (10–30 µM), cells were treated individually with the respective drugs or in combination with palbociclib for 3 days. For 7-day treatments, the media was replaced with fresh reagents every 3–4 days.

In the sequential mode, PM cells were pre-treated with 0.25 µM palbociclib for 3 days, after which the treatment was switched to the indicated concentrations of the respective BH3 mimetic for 4 more days. For sequential treatments with palbociclib and the SRC inhibitor dasatinib or Metformin, cells were pre-treated with 0.25 µM palbociclib for 7 days, then switched to dasatinib or metformin for 3 more days. At the end of treatments, cells were fixed with 4% formaldehyde and stained with crystal violet. The absorbance of dissolved crystal violet measured cell viability. Cell counts were measured using Fiji (ImageJ) software from three randomly selected images per well.

### Phospho-kinase proteome array

To visualise proteins activated by phosphorylation after palbociclib treatment, H28, MSTO-211H and ICO_MPM3 cells were treated with 0 (Control) or 0.25 µM palbociclib for 72 h and subjected to a phospho-kinase antibody array with Proteome Profiler Human Phospho-Kinase Array Kit (ARY003C R&D Biosystems). The protocol was followed as provided by the supplier.

### Single cell sorting of live senescent cells

H28 and ICO_MPM2 cells were treated with palbociclib for 15 days with drug replenishment every 3–4 days. On day 15, cells were harvested and stained with DAPI, DRAQ5 and SA-β-Gal fluorescent substrate and sorted into four groups of Small (S), Medium (M), Large/extra Large β-Gal low cells (L/XL-β-Gal^Low^) and Large/extra Large β-Gal High cells (L/XL-β-Gal^High^) based on size and β-Gal activity. The sorted cells were seeded into 48-well plates in fresh drug-free media, and regrowth was observed for a week. The cells were then counted using flow cytometry to quantify regrowth. PM cells treated with cisplatin for 3 days and allowed to remain in drug-free media for 12 more days were used as a positive control for senescent cells and were sorted together with palbociclib-treated cells on day 15. Live cell sorting was facilitated by the FACS Discover S8 cell sorter with CellView™ image-guided technology (BD Biosciences, San Jose, CA, USA) provided by the Flow cytometry core facility at PRBB, Barcelona. The gating strategy and the FSC range used to determine cell subpopulations are shown in Supplementary Fig. 7(A–H).

### Immunostaining for γH2AX and p21 and flow cytometry analysis

PM cells were treated with cisplatin or palbociclib, or subjected to drug removal, as described above. After treatment, live cells were harvested, washed with PBS, and fixed with 4% formaldehyde for 20 min at room temperature. Cells were then permeabilised with ice-cold methanol for 10 min. Cells were then incubated with primary antibodies against phospho-γH2AX and p21 according to the manufacturer’s flow cytometry protocol. Secondary staining was performed with Alexa Fluor 488-conjugated antibody for phospho-γH2AX and Alexa Fluor 546-conjugated antibody for p21 for 30 min in the dark. Stained cells were washed and resuspended in PBS for flow cytometry acquisition. Data were acquired on a CytoFLEX flow cytometer (Beckman Coulter, Brea, CA, USA). Singlets were gated, and the total median fluorescence intensity (MFI) of each channel was used as a measure of protein expression. Cytometry data were analysed using CytExpert software (Beckman Coulter).

### Statistical analysis

Data are presented as mean ± SD or mean ± SEM, as indicated. When comparing data from multiple groups, two-way ANOVA was used with Šídák’s or Tukey’s multiple comparisons tests where applicable. Sample size and replicates were chosen based on previous studies and are indicated for each method described above. When needed, outliers were removed using the find outliers functionality in GraphPad Prism. Statistical analysis was performed with GraphPad Prism 5.0 (GraphPad Software, San Diego, CA, USA). A two-tailed *P* < 0.05 was considered statistically significant.

## Supplementary information

Supplementary information

Original Western blots

## Data Availability

All the data that support the findings of this study are available from the corresponding author upon reasonable request.
