# Supplementary material for: Pseudo-senescence induced by palbociclib does not sensitise pleural mesothelioma cells to combinations with senolytics
Source: Cell Death Dis. 2026 Apr 10;17(1):388. doi: 10.1038/s41419-026-08696-z (PMC13076679; doi:10.1038/s41419-026-08696-z)
Supplement: Supplementary file 1 — Supplementary information [file 41419_2026_8696_MOESM1_ESM.pdf]

Supplementary Figure 1: Palbociclib pre-treated cells regrow upon drug washout, and regrowing PM cells retain sensitivity upon palbociclib re-introduction.

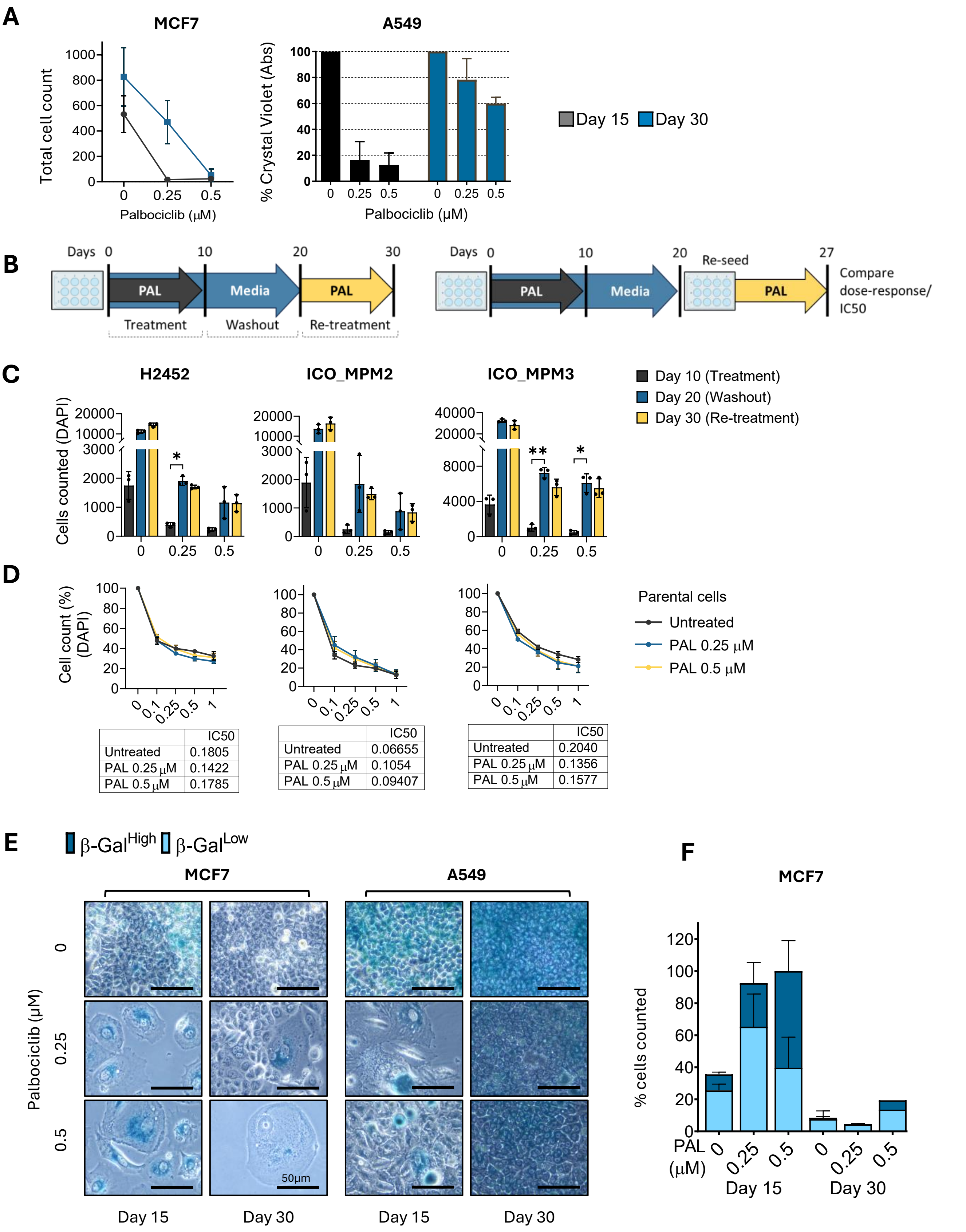

	IC50
Untreated	0.1805
PAL 0.25 μM	0.1422
PAL 0.5 μM	0.1785

	IC50
Untreated	0.06655
PAL 0.25 μM	0.1054
PAL 0.5 μM	0.09407

	IC50
Untreated	0.2040
PAL 0.25 μM	0.1356
PAL 0.5 μM	0.1577

Supplementary Figure 1: Palbociclib pre-treated cells regrow upon drug washout, and regrowing PM cells retain sensitivity upon palbociclib re-introduction.

A. Adenocarcinoma cell lines, MCF7 (breast) and A549 (Lung), were treated with palbociclib at indicated concentrations for 15 days and allowed to grow in drug-free media for 15 additional days. For MCF7 cells, the average number of cells on days 15 and 30 was determined from four random images per well after crystal violet staining. Data represent mean ± SD (N=2). For A549 cells, cell viability was measured by crystal violet absorbance on days 15 and 30. Data represented as mean ± SD (N=2).
B. PM cell lines (H2452, ICO_MPM2, ICO_MPM3) were treated with palbociclib at indicated concentrations for 10 days and allowed to grow in drug-free media for 10 additional days. On day 20, either the same cell populations were re-treated with palbociclib for a further 10 days or cells from regrowth were trypsinised and reseeded to assess the dose-response to 7-day treatment with palbociclib. On days 30 and 27, the cells were counted after DAPI staining.
C. The average number of cells on days 10, 20, and 30 was counted from six random pictures per well after DAPI staining. Data represent mean ± SD (N=3). Statistical significance determined from Two-way ANOVA comparing cell numbers on day 10 vs day 20 and on day 20 vs. day 30. *$p < 0.05$, ***$p < 0.001$, ****$p<0.0001$.
D. The dose-response curves for the percentage of PM cells counted at Day 27 after re-treatment with palbociclib at the indicated doses. Data represent mean ± SD (N=3).
E. Representative images of MCF7 and A549 cells stained for SA-β-Gal activity showing cell morphology and SA-β-Gal activity on day 15 (post-treatment) and 30 (drug removal). Images were taken at 40X.
F. The percentage of MCF7 cells showing high or low SA-β-Gal was counted from four random pictures per well. Data represent mean ± SEM (N=2).

Supplementary Fig. 2. PM cells express anti-apoptotic proteins of the Bcl-2 family.

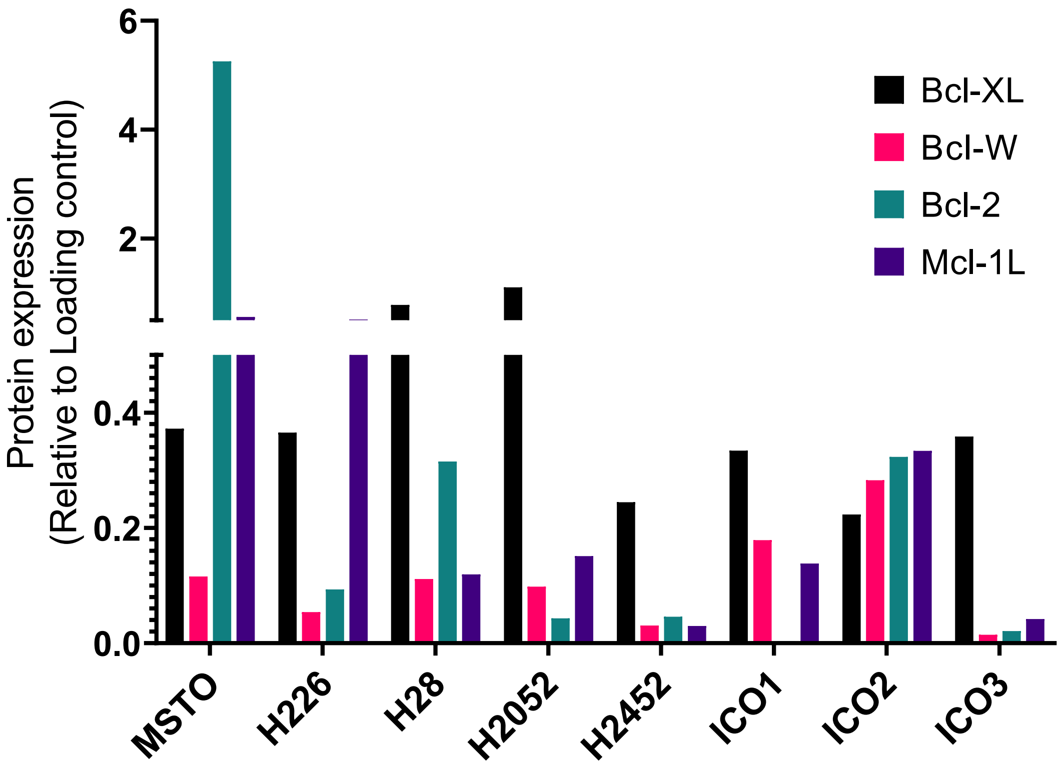

Cell lysates of commercial and patient-derived PM cell lines were probed for anti-apoptotic Bcl-xL, Bcl-W, Mcl-1 and Bcl-2. Each bar represents the mean fold change in the baseline expression of these proteins, normalised to the loading control (Actin) from at least two biological replicates. Data is represented as mean.

Supplementary Figure 3: BH3 mimetics do not synergize with palbociclib in eliminating PM cells

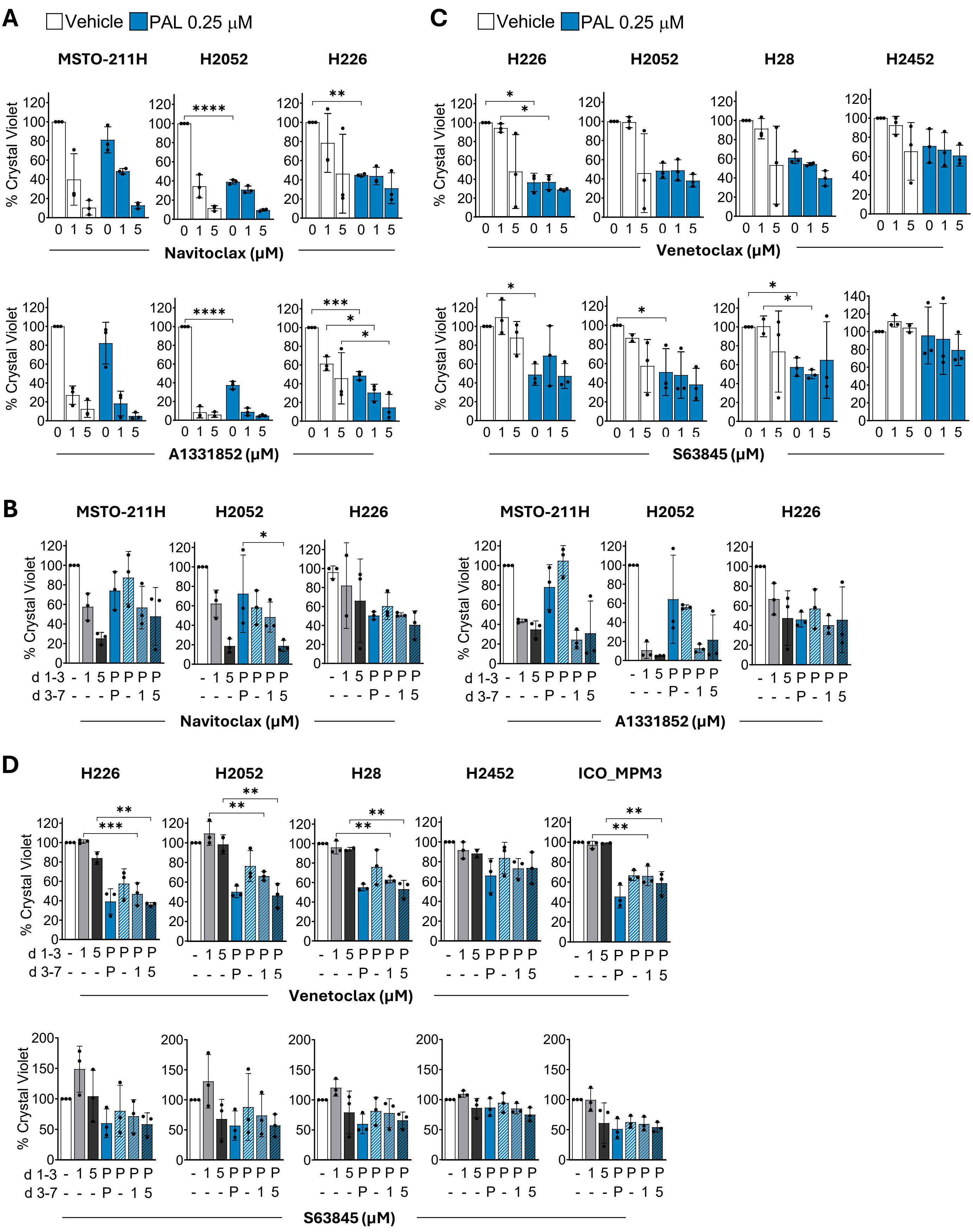

Supplementary Figure 3: BH3 mimetics do not synergise with palbociclib in eliminating PM cells

A. PM cell lines MSTO-211H, H2052 and H226 were treated with 0.25 µM palbociclib in the presence or absence of BH3-mimetics, Navitoclax (ABT-263) or A1331852 at the indicated concentrations for 7 days. Data represent the mean ± SD of the percentage absorbance at 7 days for crystal violet in each treatment, normalised to the untreated control (N=3).

B. PM cells MSTO-211H, H2052 and H226 were pre-treated with 0.25 µM palbociclib ("P") for 3 days before switching the treatment to BH3-mimetics, Navitoclax (ABT-263) or A1331852 at the indicated concentrations for 4 days more. Cells were stained with crystal violet on day 7. Data represent the mean ± SD of the percentage absorbance on day 7 from crystal violet, normalised to the untreated control (N=3).

C. H226, H2052, H28 and H2452 cells were treated with 0.25 µM palbociclib in the presence or absence of BH3-mimetics Venetoclax (A-199) or S63845 at the indicated concentrations for 7 days. Data represent the mean ± SD of the percentage absorbance at 7 days for crystal violet in each treatment, normalised to the untreated control (N=3). For both (A) and (B), statistical significance was determined from two-way ANOVA with multiple comparisons of individual treatments to their respective combinations (*p < 0.05, **p < 0.01, ***p < 0.001, ****p < 0.0001).

D. H226, H2052, H28, H2452 and ICO_MPM3 cells were pre-treated with 0.25 µM palbociclib ("P") for 3 days before switching the treatment to BH3-mimetics, Venetoclax (A-199) or S63845 at the indicated concentrations for 4 days more. Cells were stained with crystal violet on day 7. Data represent the mean ± SD of the percentage absorbance on day 7 from crystal violet, normalised to the untreated control (N=3). For both (D) and (E), statistical significance was determined from ordinary one-way ANOVA with multiple comparisons (*p < 0.05, **p < 0.01, ***p < 0.001, ***p < 0.0001).

Supplementary Figure 4: Adaptive response to palbociclib guided other combination treatments

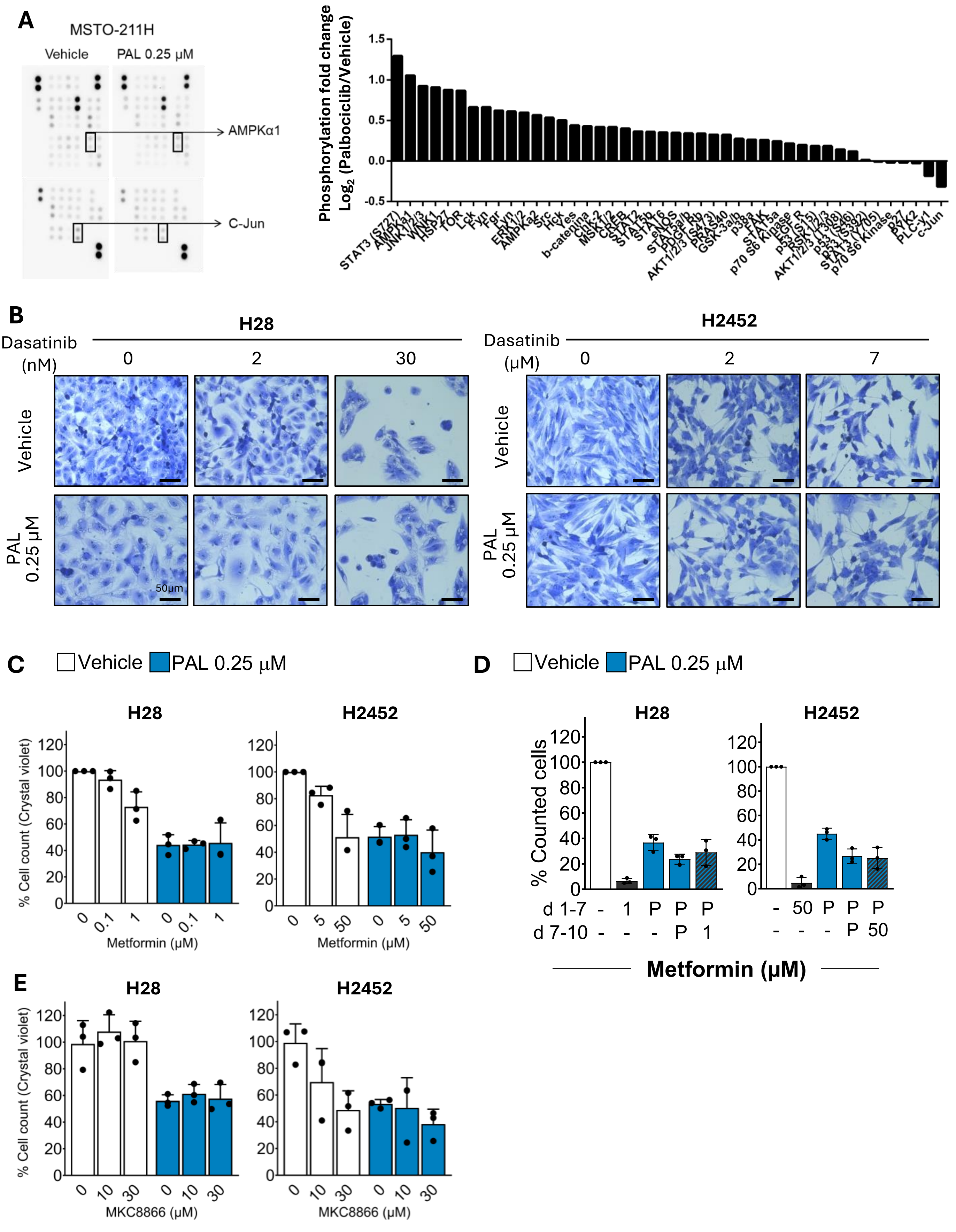

Supplementary Figure 4: Adaptive response to palbociclib guided other combination treatments

A. Proteomic profile of MSTO-211H cells treated with Vehicle or palbociclib for 3 days shows the activation and upregulation of a range of phospho-kinases after palbociclib treatment.

B. Representative figures taken at 20X of H28 and H2452 cells treated with Vehicle or 0.25 μM palbociclib in the presence or absence of varying concentrations of Dasatinib (Src kinase inhibitor) for 7 days and stained with Crystal violet.

C. PM cell lines H28 and H2452 were co-treated with 0.25 μM palbociclib and varying concentrations of metformin for 3 days.

D. PM cells were pre-treated with 0.25 μM palbociclib for 7 days, then switched to Metformin/ drug-free media for 3 more days. Cells were stained with crystal violet and counted from three random images per well. Data represent the mean ± SD of the percentage of cells counted under each treatment condition, normalised to the untreated control. (N=3).

E. PM cell lines H28 and H2452 were co-treated with 0.25 μM palbociclib and varying concentrations of MKC8866 (IRE1α inhibitor) for 3 days. Cell count was determined from three random pictures per well following crystal violet staining. Data represent the mean ± SD of the percentage of cells counted under each treatment condition, normalised to the untreated control (N=3).

Supplementary Figure 5:

High doses of Cisplatin engage permanent cell cycle arrest with a senescence phenotype in PM cells and MCF7 cells.

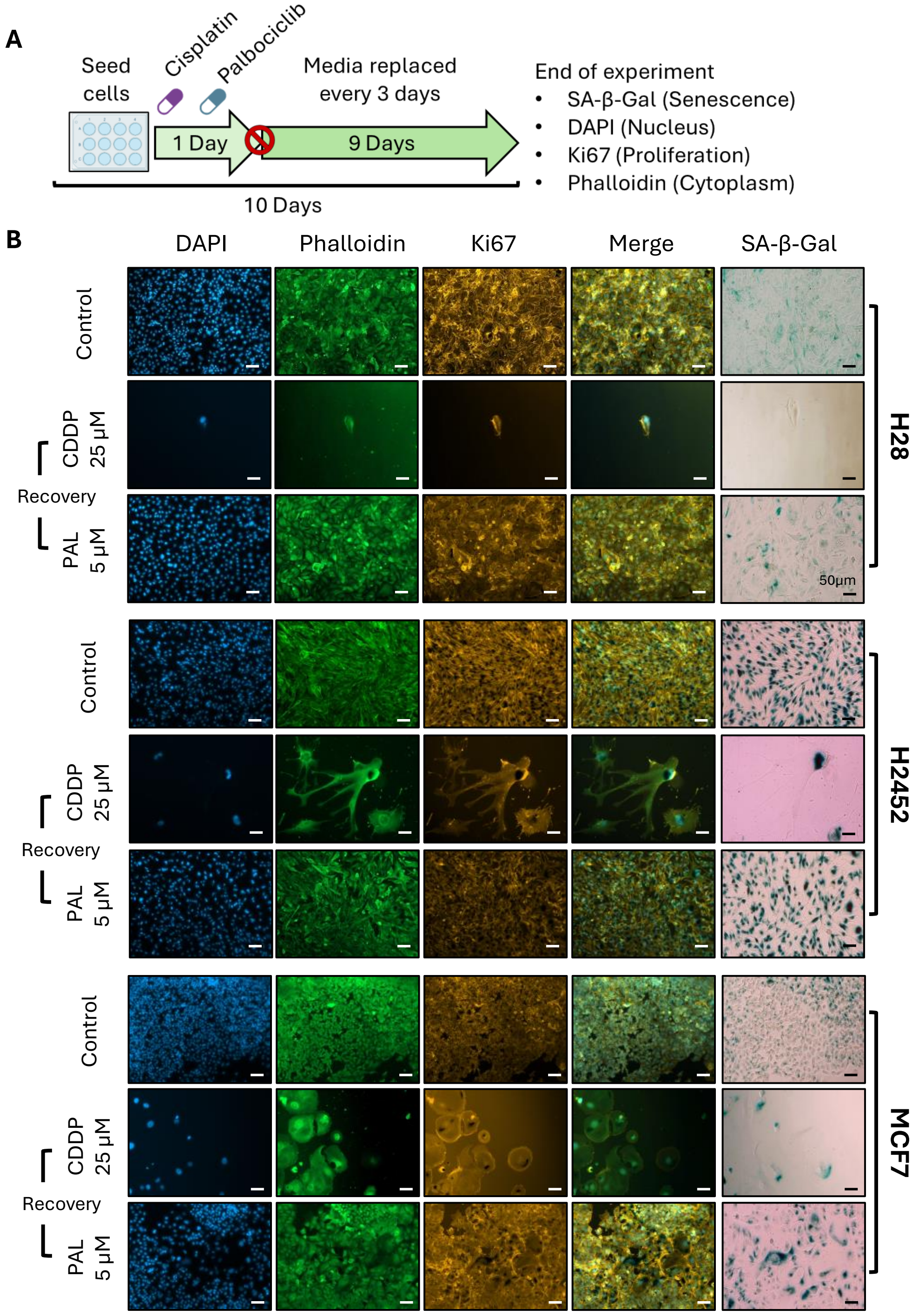

A. H28, H2452 and MCF7 cells were treated with high doses of palbociclib or Cisplatin for 1 day before switching to drug-free media for 9 more days. On day 10, they were stained for β-Galactosidase activity and co-stained with DAPI, Phalloidin and Ki67.

B. Representative images of PM cells and MCF7 co-stained with DAPI, Ki67, Phalloidin and X-Gal substrate showing β-Gal activity and varied morphological features after pre-treatment with palbociclib/ cisplatin. Images were taken at 10X on day 10.

Supplementary Figure 6: Cisplatin exerts a stronger cytostatic effect in PM cells than palbociclib.

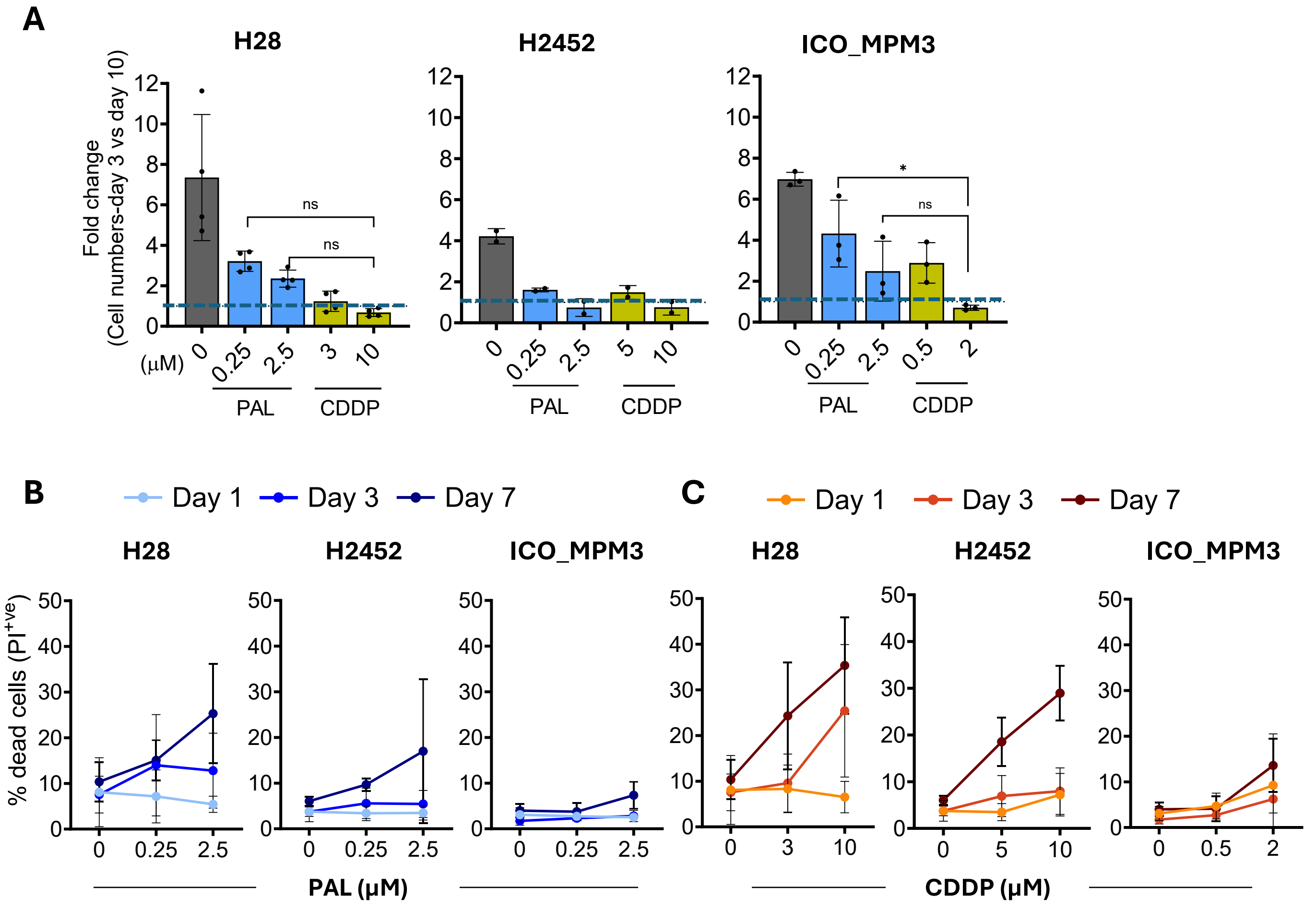

A. PM cell lines H28, H2452, and ICO_MPM3 were treated with the indicated doses of palbociclib (PAL) or cisplatin (CDDP) for 3 days, then switched to drug-free media for 7 more days. At days 3 and 10, cell nuclei were counted from 3 random pictures per well following DAPI staining. The difference in cell growth is represented as a fold change between cells counted on Day 3 Vs Day 10. A fold change >1 is considered regrowth. Data represented as mean ± SD (N=4 for H28; N=2 for H2452 and N=3 for ICO_MPM3). One-way ANOVA with Tukey´s multiple comparisons were used to determine p values (*p < 0.05, **p < 0.01, ***p < 0.001).

B. H28, H2452 and ICO_MPM3 cells were treated with palbociclib (PAL) at indicated doses for 1, 3 and 7 days. At all time points, the percentage of dead cells was measured by PI staining with flow cytometry. Data represented as mean ± SD (N=3).

C. H28, H2452 and ICO_MPM3 cells were treated with cisplatin (CDDP) at indicated doses for 1, 3 and 7 days. At all time points, the percentage of dead cells was measured by PI staining with flow cytometry. Data represented as mean ± SD (N=3).

Supplementary Figure 7

Untreated cells- ICO_MPM2- Senescent cell sorting

A B C D E F

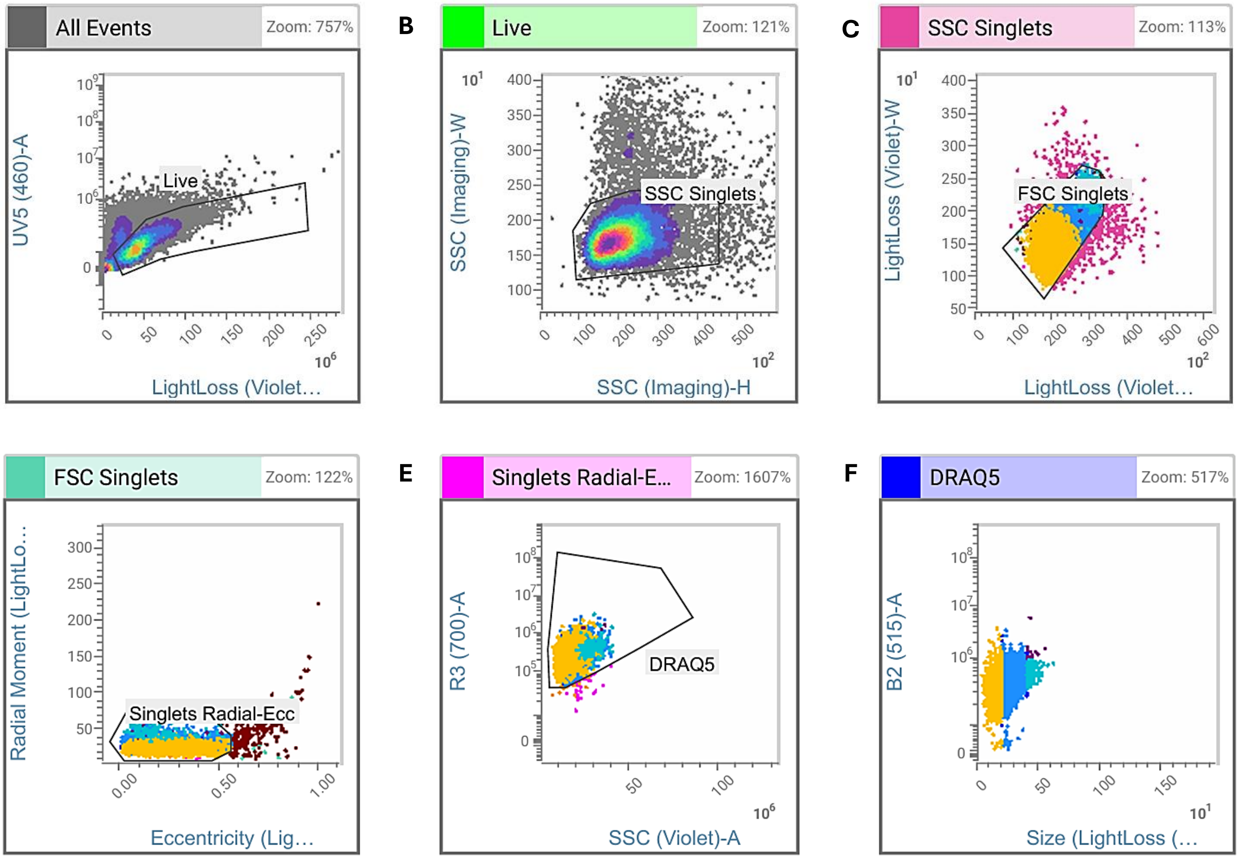

G

Cell size	FSC range
S	<20
M	20-40
L	40-80
XL	>80

S
M
L/XL-β-Gal[Low]
L/XL-β-Gal[High]

H

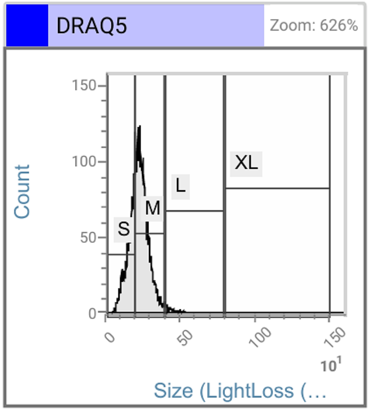

I

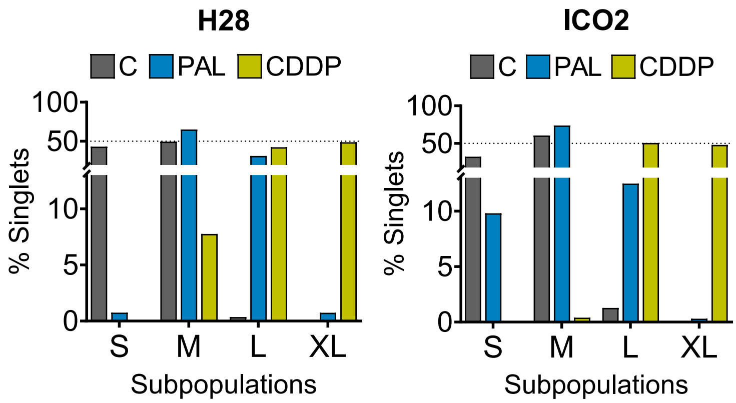

J

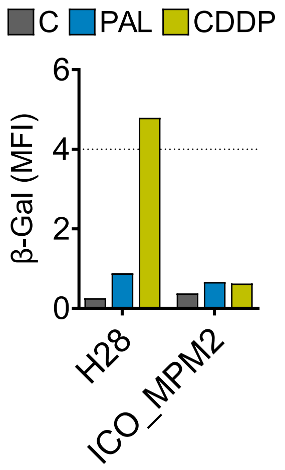

Supplementary Figure 8:

The gating strategy for senescent live-cell sorting.

A-F: Cytograms from ICO_MPM2 cells in untreated conditions as an example of cell sorting experiment.

A. Live cells were selected by gating on light loss (FSC) vs UV (460)-A to exclude debris and dead cells positive for DAPI.
B. From the live cell population, singlets were gated based on SSC-H vs SSC-W.
C. From the SSC-singlets population, singlets were filtered once more by gating light loss (FSC)-H vs light loss (FSC)-W.
D. From the FSC-singlets population, cells were selected for eccentricity and radial moment to gate for cells with intact cell membrane structure and centred fluorescent intensity.
E. From gate D, nucleated cells were selected by gating cells positive for DRAQ5.
F. Distribution of DRAQ5-positive events in the FITC-channel (Y axis) that represents SA-β-Gal activity, and size-light loss in the X axis. This is a imaging parameter which is a more accurate measure for cell size than FSC light loss.
G. From gated regions shown in F, subpopulations of interest are gated based on size and β-gal substrate, and labeled Small (S), Medium (M), Large (L), and extra-large (XL) cells depending on the FSC range used to categorise them. The largest cells (if present) in a subpopulation (L/XL) were further separated as β-Gal^{Low} and β-Gal^{High} based on SA-β-Gal activity.
H. An example of the distribution of subpopulations of interest based on size-light loss (FSC).
I. Percentage of singlets in each categorised sub-population of H28 and ICO_MPM2 cells after treatment with palbociclib or cisplatin.
J. The median fluorescent intensity (MFI) of SA-β-Gal activity in H28 and ICO_MPM2 cells treated with palbociclib or cisplatin.

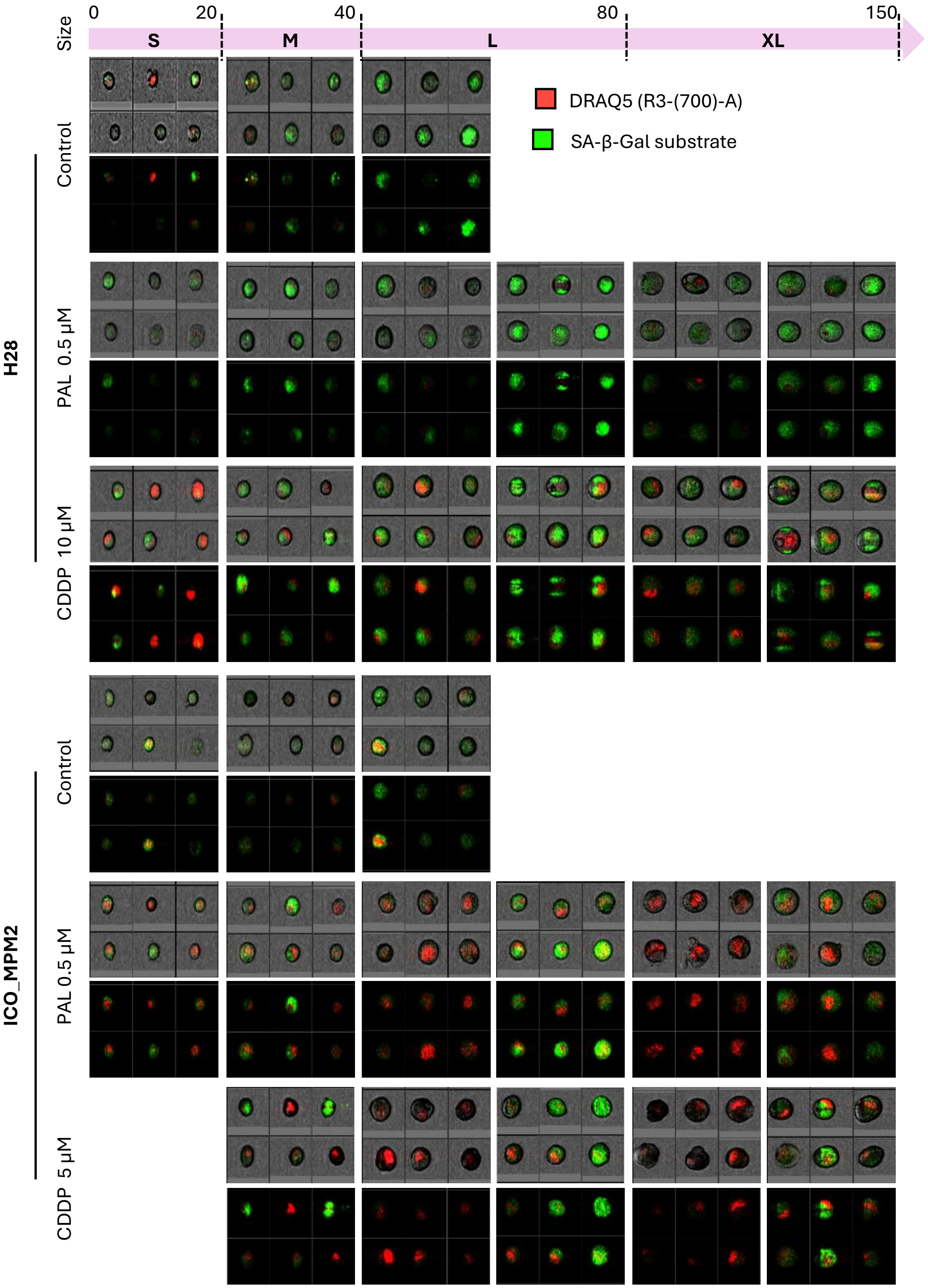

Supplementary Figure 8: Representative pictures for PM cells categorised into subpopulations stained with DRAQ5 (red) and SA-β-Gal substrate (green) post-treatment with palbociclib or cisplatin.

Upper panels indicate all channels (ightloss (Imaging)/ DRAQ5/ SA-β-Gal substrate) and lower panels indicate only DRAQ5/ SA-β-Gal substrate.
