# Supplementary material for: Pseudo-senescence induced by palbociclib does not sensitise pleural mesothelioma cells to combinations with senolytics
Source: Cell Death Dis. 2026 Apr 10;17(1):388. doi: 10.1038/s41419-026-08696-z (PMC13076679; doi:10.1038/s41419-026-08696-z)

Original Western Blot

Figure 2: MPM cells express anti-apoptotic proteins of the Bcl-2 family in baseline conditions.

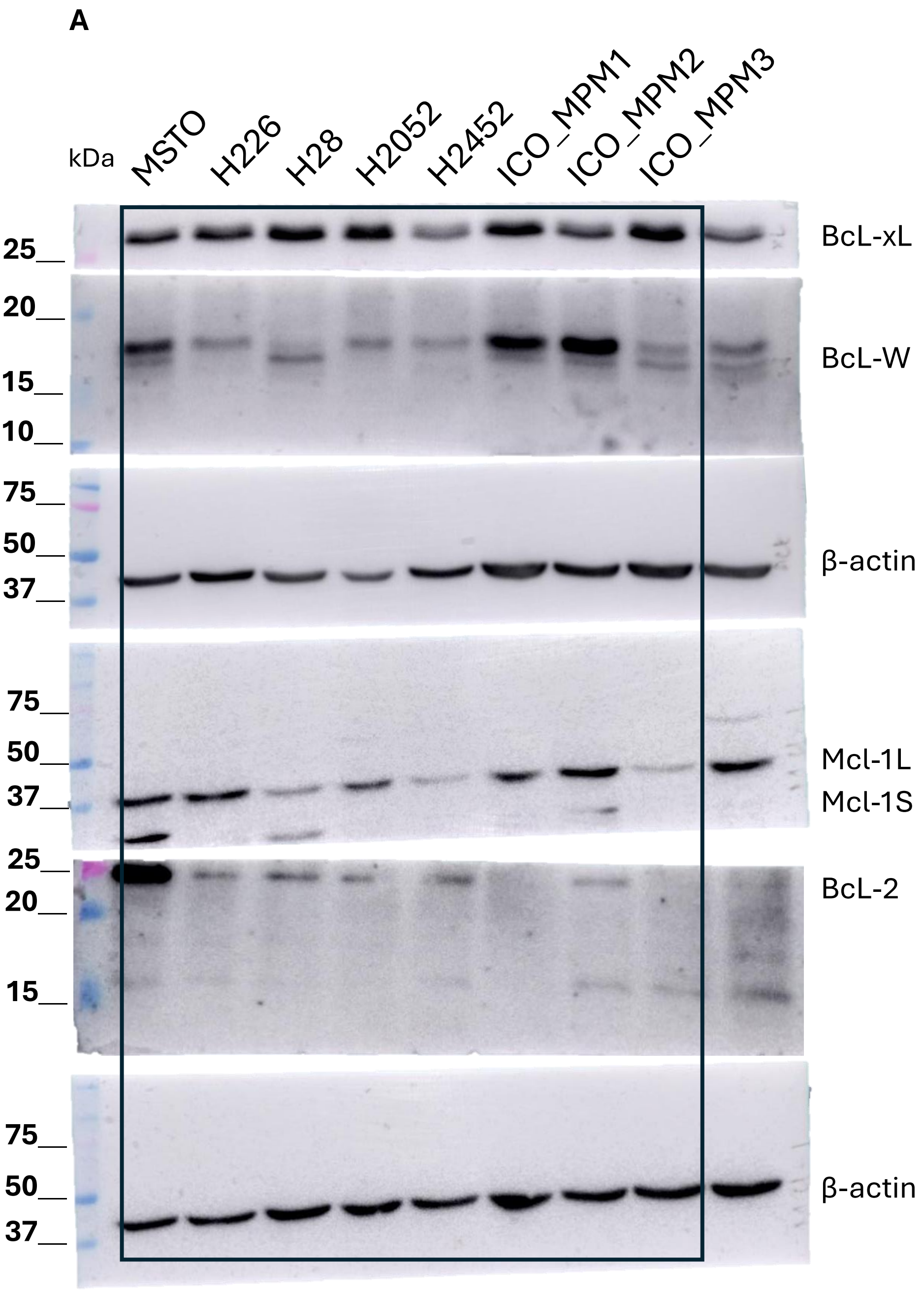

Original western blots

Original Western Blot

Figure 2: MPM cells express anti-apoptotic proteins of the Bcl-2 family in baseline conditions.

B

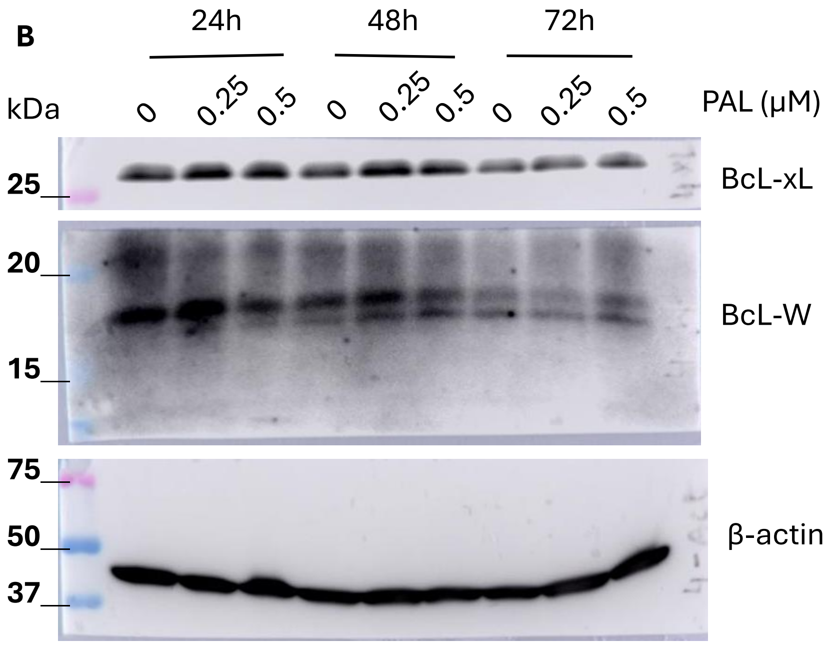

D

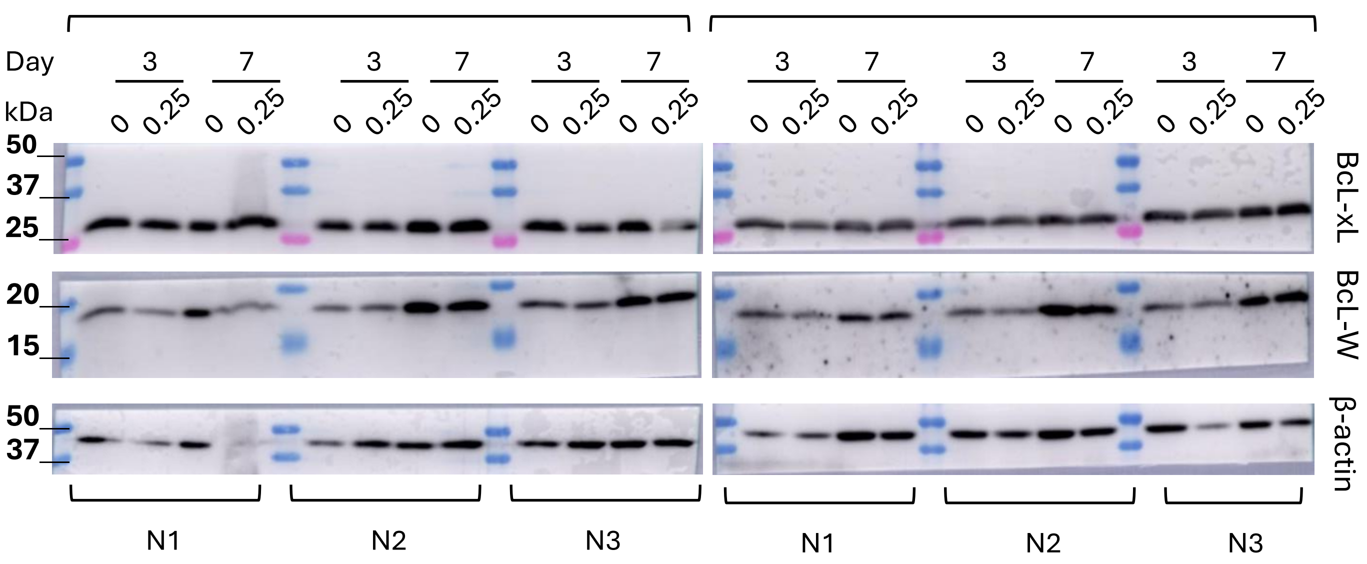

Supplement: Supplementary file 2 — Original Western blots [file 41419_2026_8696_MOESM2_ESM.pdf]
